# Age differences in the neural basis of decision-making under uncertainty

**DOI:** 10.3758/s13415-022-01060-6

**Published:** 2023-03-08

**Authors:** Loreen Tisdall, Rui Mata

**Affiliations:** Center for Cognitive and Decision Sciences, University of Basel, Missionsstrasse 60-62, 4055 Basel, Switzerland

**Keywords:** Aging, Brain, Uncertainty, Risk, Ambiguity, BART, Temporal discounting

## Abstract

Humans globally are reaping the benefits of longer lives. Yet, longer life spans also require engaging with consequential but often uncertain decisions well into old age. Previous research has yielded mixed findings with regards to life span differences in how individuals make decisions under uncertainty. One factor contributing to the heterogeneity of findings is the diversity of paradigms that cover different aspects of uncertainty and tap into different cognitive and affective mechanisms. In this study, 175 participants (53.14% females, mean age = 44.9 years, SD = 19.0, age range = 16 to 81) completed functional neuroimaging versions of two prominent paradigms in this area, the Balloon Analogue Risk Task and the Delay Discounting Task. Guided by neurobiological accounts of age-related changes in decision-making under uncertainty, we examined age effects on neural activation differences in decision-relevant brain structures, and compared these across multiple contrasts for the two paradigms using specification curve analysis. In line with theoretical predictions, we find age differences in nucleus accumbens, anterior insula, and medial prefrontal cortex, but the results vary across paradigm and contrasts. Our results are in line with existing theories of age differences in decision making and their neural substrates, yet also suggest the need for a broader research agenda that considers how both individual and task characteristics determine the way humans deal with uncertainty.

**Supplementary Information:**

The online version contains supplementary material available at 10.3758/s13415-022-01060-610.3758/s13415-022-01060-6.

Uncertainty can come in many guises, be linked to many different sources (e.g., self vs. others), and refer to different properties of choice options, such as their magnitude or probability, which in turn can be static or change over time (Dhami & Mandel, 2022; Meder et al., 2013). Given the vast possible forms of uncertainty we face, the proliferation of approaches and paradigms used in the psychological sciences to understand age differences in dealing with uncertainty is not surprising; neither are the conflicting results concerning the existence or magnitude of age effects in the associated paradigms (Mata et al., 2011; Best & Charness, 2015; Seaman et al., 2022; Lighthall, 2020) and their neural bases (Tannou et al., 2021; Samanez-Larkin & Knutson, 2015; Lighthall, 2020). In this work, we contribute to this area of research by reporting results from a neuroimaging study designed to assess age differences in the neural basis of decision-making under uncertainty.

In what follows, we first engage with the concept of uncertainty, including varying definitions and paradigms used for assessment of individual and age differences in dealing with uncertainty. Second, we provide an overview of past empirical work examining the neural basis of decision-making under uncertainty. Third, we review the current empirical literature on the link between aging and decision-making under uncertainty. Fourth, and finally, we describe our current study, which aims to offer an empirical contribution towards assessing age differences in decisions under uncertainty.

## Uncertainty: definition and scope

Many researchers have often used the terms uncertainty, risk, and ambiguity interchangeably (Dhami & Mandel, 2022), but more specific distinctions between these concepts can be made. Risk can be defined in many ways (Aven, 2012), yet a common understanding of risky decision-making refers to decisions that involve, at a minimum, uncertain gains and losses (Schonberg et al., 2011). How individuals come to know about prospective gains, losses, and their respective probabilities, however, can vary. In the lab, gamified lotteries are popular tools to assess individuals’ risk preference, as these allow researchers to exert full experimental control over the information provided. For example, crucial information about magnitudes and probabilities might be described from the outset, or might have to be learned through (repeated) experience. The crucial aspect of decision-making under risk, however, is that it refers to situations in which outcomes and their probabilities are known. In contrast, ambiguity or “Knightian uncertainty” describes situations in which only the outcomes, but not their probabilities, are known (Meder et al., 2013; Tymula et al., 2013; Tymula et al., 2012). As such, relative to risk, ambiguity might be perceived as entailing even more uncertainty (Wu et al., 2021).

Some behavioral paradigms have natural ways of offering distinctions between risk and ambiguity. For example, when monetary gambles are presented descriptively to participants (e.g., the magnitude and probabilities of choice options are stated explicitly), manipulations of risk are easily achieved. However, in some cases, participants need to learn about options and probabilities over time, which leads to a continuum between risk and ambiguity, because the representation of ambiguity can change into a representation of risk as a function of each individual’s learning experience. Importantly, some researchers have proposed that age differences in learning can partly account for age patterns in dealing with decision-making under uncertainty in such scenarios, making it particularly interesting to examine paradigms involving such components in order to understand age differences in dealing with uncertainty (Frey et al., 2015; Henninger et al., 2010).

In the field of economics, it is also common to make a distinction between preferences for risk, ambiguity, and time, because, in principle, economic agents can be given different types of choices involving trade-offs about option characteristics, such as their magnitudes, probabilities, and time of delivery, independently. However, some have proposed that there is an inherent uncertainty associated with making decisions about the future, leading to a direct link between risk, ambiguity, and time preferences (Epper et al., 2011; Cohen et al., 2020). Importantly, some aging theories have proposed that uncertainty about the future associated with older adults’ limited time horizon is an important factor in determining age effects in temporal discounting (Seaman et al., 2022). Thus, it is possible that paradigms involving risk, ambiguity, and temporal trade-offs all tap into individuals’ processing of, and attitudes toward, uncertainty.

We propose a research agenda that considers the many guises in which uncertainty can be presented and investigates age differences in the underlying cognitive and affective processes by assessing the neural basis of decisions under uncertainty.

## Neural correlates of decision-making under uncertainty

In this section, we provide a brief overview of studies which have used functional neuroimaging methods to elucidate the brain regions involved in decision-making under uncertainty. In particular, we review studies that have investigated risk, ambiguity, or temporal preferences, and which can form a basis for understanding how aging can affect the associated decision processes.

Some theories have proposed a neural risk matrix to account for decision-making under risk (Knutson & Huettel, 2015), which includes brain regions that promote (ventral striatum), inhibit (anterior insula), and modulate (dorsomedial prefrontal cortex, also referred to as the anterior cingulate cortex) risky choice. Ambiguity, in turn, has been suggested to elicit (stronger) activation differences in a range of neural regions, including amygdala, inferior and posterior parietal lobe, anterior insula, orbitofrontal cortex, and inferior frontal gyrus (Wu et al., 2021). Recent meta-analytic work confirms the view that risk and ambiguity evidence both overlapping and unique neural substrates (Wu et al., 2021). In particular, risk and ambiguity do indeed share activation differences in anterior insula, which has led some to suggest that the anterior insular cortex is a key region for processing uncertainty (Wu et al., 2021). However, whereas decision-making under risk relies on ventral striatum and dorsomedial prefrontal cortex (i.e., anterior cingulate cortex) activation (Knutson & Huettel, 2015), ambiguity relies on dorsolateral prefrontal cortex and inferior parietal lobe (Wu et al., 2021). Interestingly, it has been argued that the additional finding of increased involvement of anterior insula activation in decision-making under ambiguity relative to decision-making under risk maps onto the proposal of uncertainty being more pronounced in ambiguity relative to risk (Wu et al., 2021).

Temporal preference refers to an individual’s tendency to devalue future options, with high delay discounting describing the tendency to devalue future options at a steep(er) rate. Multiple aspects influence an individual’s preference concerning temporally delayed options, such that higher discounting (i.e., more devaluation), for example, can stem from heightened sensitivity to anticipated present rewards, lower sensitivity to anticipated future rewards, or the suboptimal integration of present and future rewards into an overall subjective value signal. From a neurobiological standpoint, each computation is associated with specific neural correlates (Samanez-Larkin & Knutson, 2015; McClure et al., 2004; van den Bos et al., 2014). A recent review of the empirical literature on the neural basis of temporal preference (Frost & McNaughton, 2017) found evidence for the involvement of primarily striatal and (pre)frontal cortical regions.

To isolate the neural basis of uncertainty in delay discounting, different methodological approaches can be used. One approach involves the calculation of an integrated subjective value for each offer based on individuals’ choices, and the tracking of this value signal across the brain (Seaman et al., 2018). Alternatively, uncertainty could be captured by focusing on the neural representation of uncertainty related to the temporal features of various trade-offs between choice options. For example, one can examine the neural substrates of immediacy; that is, examine the neural functional differences between the processing of trade-offs with and without immediate options, under the assumption that an immediate option involves reduced uncertainty. Research along these lines has found decision-making relevant activation differences for immediate relative to delayed options in ventral striatum, medial prefrontal and medial orbitofrontal cortex (McClure et al., 2004; Frost & McNaughton, 2017). Second, one can consider reward delays, the logic being that longer delays evoke higher uncertainty relative to shorter delays. Empirical findings suggest increased activation in ventral striatum and putamen for shorter relative to longer delays (Wu et al., 2021).

All in all, empirical results across risk, ambiguity, and time preferences suggest that although anterior insula activation could code uncertainty in decisions under risk and ambiguity, when it comes to temporal trade-offs, uncertainty may be coded as a relative activation difference in reward valuation and/or integration regions, such as nucleus accumbens and the ventral medial prefrontal cortex. Decision-making under uncertainty, whether in relation to risk, ambiguity, or temporal trade-offs, thus seems to rely on both common and unique brain circuitry. Yet, to what extent age effects in decision-making under uncertainty are driven by changes in single or several brain regions remains to be determined.

### Age differences in decision-making under uncertainty

Empirical results on age differences in dealing with risk are patently mixed, with meta-analyses and qualitative reviews suggesting that age effects vary considerably across measures (Mata et al., 2011; Best & Charness, 2015; König, 2021). Overall, self-report measures suggest age-related declines in the propensity to take risks (König, 2021) but task-based results are more heterogeneous, with meta-analyses suggesting decreased risk taking with age for gains but not losses (Best & Charness, 2015). There is overall less work on situations involving ambiguity, when the probability of options is not described (Tymula et al., 2013) or needs to be learned from experience (Frey et al., 2015; Frey et al., 2021; Mata et al., 2011). One paradigm that has received considerable attention is the Balloon Analogue Risk Task (BART; Lejuez et al.,^2002^) but the results concerning age differences are also mixed: Although some studies find older adults less risk-seeking relative to younger adults (Grover, 2021; Henninger et al., 2010; Koscielniak et al., 2016; Rolison et al., 2012; Sproten et al., 2018; Wilson et al., 2021), a number of studies find this result in only some conditions (Mamerow et al., 2016; Schulman et al., 2021), report no evidence of behavioral effects of age (Kim et al., 2022; McCleskey, 2021; Yu et al., 2016), or find that older-adults are more risk-seeking relative to younger adults (Cavanagh et al., 2012). Finally, concerning age effects on temporal preferences, a recent meta-analytic synthesis suggests overall no effect of age in delay discounting tasks (Seaman et al., 2022).

As it stands, there is limited knowledge concerning age differences in many paradigms involving decision-making under uncertainty. For example, to our knowledge only one study has investigated age differences in the neural basis using the BART (Yu et al., 2016). Yu and colleagues (Yu et al., 2016) found evidence that suggests preservation of value signals for processing of gains and losses but age differences in ventromedial prefrontal cortex potentially reflecting age differences in information integration. Concerning temporal preferences, a few neuroimaging studies examining adult age differences suggest activation differences (Samanez-Larkin et al., 2011; Sasse et al., 2017). For example, one study found younger adults showed more nucleus accumbens activation for present than future rewards relative to older adults (Eppinger et al., 2012), which could signal age differences in the valuation of uncertainty associated with future outcomes. However, more recent efforts aimed at isolating age effects on the neural representation of subjective value found no differences for temporal preferences (Seaman et al., 2018).

There is so far relatively little in the way of a quantitative synthesis of age differences in the neural basis of decision-making under uncertainty (Tannou et al., 2021), but existing frameworks and qualitative reviews suggest that aging may be associated with changes in the anticipation of gains and losses, as well as the integration of different sources of information in decisions made under uncertainty (Samanez-Larkin & Knutson, 2015; Lighthall, 2020). Crucially, age-related functional and structural brain changes are not global but specific to particular neural regions (Sowell et al., 2004; Cabeza, 2002), some of which are highly relevant for explaining age effects on decision making. Exemplifying this approach is the Affect-Integration-Motivation (AIM) framework (Samanez-Larkin & Knutson, 2015), an empirically derived neurobiological framework. By breaking the decision-making process down into various sequential, hierarchically arranged processes, and identifying the respective neural correlates, AIM stipulates several pathways for how age-related anatomical and functional change may lead to age-related differences during varying decision-making stages (Frazier et al., 2019; Samanez-Larkin & Knutson, 2015), including anticipated reward-related activity in nucleus accumbens, anticipated loss-related activity in anterior insula, integrative processes subserved by activation in prefrontal cortices, and, in situations involving learning, the thalamus.

All in all, the majority of aging studies reviewed above have focused on single tasks, involved relatively small sample sizes and extreme-group comparisons, did not specifically target theory-relevant brain regions (Samanez-Larkin & Knutson, 2015), nor have they adopted a systematic approach to controlling for theoretically relevant covariates (for a critique of the past literature see Mata et al., 2011; Seaman et al., 2022; Frey et al., 2021). As a consequence, we still know relatively little about the robustness of age differences in behavioral and neural patterns associated with many of these measures.

### Overview of the current study

The review above highlights a number of key limitations of the current literature. In particular, it has become clear that there is a dearth of studies examining adult age differences using multiple paradigms that allow to distinguish several cognitive and affective processes underlying the processing of uncertainty. We aim to contribute to this effort by conducting an empirical assessment of a relatively large age-heterogeneous sample aged 16 to 81 using two paradigms, the BART and the Delay Discounting Task. Informed by the AIM framework (Samanez-Larkin & Knutson, 2015), we do so by analyzing activation differences in an *a priori* selected set of neural regions key for decision-making under uncertainty, and by exploring a number of key contrasts that tap into different aspects of uncertainty. Specifically, in the BART, we test the importance of using average and parametric contrasts that have been previously considered in the literature (Yu et al., 2016) as well as a novel contrast comparing different (i.e., linear and exponential) reward functions that create varying values of uncertainty. In the delay discounting task, we consider both delay and immediacy contrasts previously used in the literature (Frost & McNaughton, 2017). Furthermore, we do so in a systematic, exhaustive fashion using specification curve analysis (Simonsohn et al., 2020; Rohrer et al., 2017), which allows us to (a) simultaneously explore the effect of age on various behavioral and neural operationalizations of decision-making under uncertainty while (b) assessing and controlling for the role of theoretically relevant covariates shown to impact decision-making under uncertainty, including gender, education, income, and cognitive ability (Frey et al., 2021). We thus hope to contribute to a more systematic assessment of age differences in the neural basis of decision-making under uncertainty.

## Method

The current analyses are based on data collected as part of a larger multi-session research study to examine age effects on multiple indicators of risk preference, impulsivity, and low self-control (preprint available via the Open Science Framework, https://osf.io/uj359), for which we preregistered the analyses (preregistration available via AsPredicted.com, https://aspredicted.org/98R_QYR). We did not preregister the current analyses, which are exploratory in nature and extend the pre-registered ones by focusing on the neural representation of uncertainty across several brain regions and task contrasts and use a multiverse analytic approach to exhaustively examine associations between these different operationalizations of uncertainty and age. To address the current research questions, we conducted original analyses of the functional neuroimaging data collected from two behavioral tasks featuring different implementations of uncertainty. The original study was reviewed and approved by the Ethikkommission Nordwest- und Zentralschweiz EKNZ (EKNZ BASEC 2015-00094). We complied with all regulations and ethical guidelines during the research. Prior to participating in the study, all participants received written as well as verbal study materials and were required to give written informed consent to their participation in the research. All participants were paid for their participation in the study [15 Swiss Francs per hour of participation, approximately 98 Swiss Francs ($\sim $98 USD) for participation in all sessions of the larger study] and were further paid in cash any additional earnings from the incentive-compatible behavioral measures completed (e.g., as part of the tasks completed during the neuroimaging session).

### Participants

For the current analyses, we started with 189 participants who were recruited for the neuroimaging session (52% female, mean age = 45 years, SD = 19) as part of the larger original study. Ten of the 189 participants had to be excluded from all further analyses because they had no or incomplete fMRI data (mainly because only one paradigm was completed due to time constraints in the MRI facility). Data from a further two participants had to be excluded after (partial) data collection was complete because of faulty equipment and another two participants were excluded because they exhibited too much head motion (see section on neuroimaging data preprocessing for further details). After exclusions, we included an effective sample of 175 individuals [93 (53.14%) females, mean age = 44.88 years, SD = 19.02, age range = 16.15 to 81.38 years] in all our analyses. A detailed characterization of the study sample with regards to demographic and sociodemographic variables is presented in Fig. A1. For further information, including power analyses, participant recruitment and screening, see Appendix A.

### Materials

**Fig. 1:**
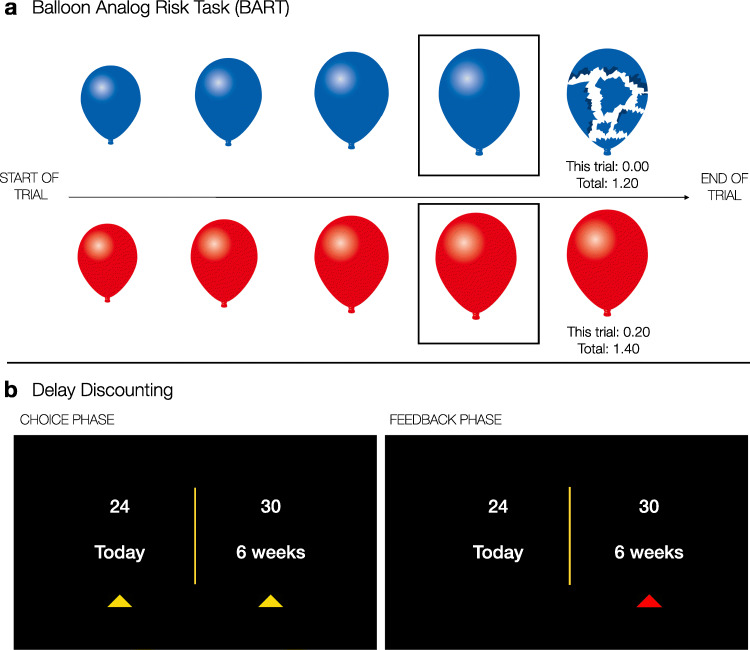
Schematic of behavioral tasks completed during the neuroimaging session. **(a)** Balloon Analogue Risk Task (BART). Top row: example of an explosion trial; bottom row: example of a cash-out trial. The frame around the second-to-last balloon did not appear on participants’ screens but was added to this schematic to highlight a pump decision leading to an explosion (top row, blue balloons) versus a cash-out decision that saves the accumulated earnings (bottom row, red balloons). **(b)** Delay Discounting Task

To capture task-related neural activation differences, participants completed two commonly used behavioral tasks involving different aspects of uncertainty inside the MRI scanner, namely the Balloon Analogue Risk Task (BART) and the Delay Discounting Task (Fig. 1). We describe each of these in more detail below.

#### BART

The BART has a long tradition in behavioral and neuroimaging research aiming to elucidate (the neural basis of) individual differences in risk taking (Lejuez et al., 2002; Rao et al., 2008; Schonberg et al., 2012; Helfinstein et al., 2014; Tisdall et al., 2020). Age effects have also been studied with the BART, at the level of both behavior (Mata et al., 2011; Rolison et al., 2012; Mamerow et al., 2016) and the brain (Yu et al., 2016; Tannou et al., 2021; Wang et al., 2022). The standard implementation of the BART involves the virtual inflation of balloons via sequential administration of pumps (i.e., button presses), yet participants are not informed about explosion points or inflation capacity. Instead, participants are required to build up a mental representation of an average explosion point through repeated choice and the experience of choice outcomes. Importantly, successful pumps (inflations which do not lead to an explosion) contribute to the accumulation of a financial reward as each successful pump is worth a certain amount of money. In contrast, if an inflation causes the balloon to explode, the earnings accumulated on the current trial are lost. Participants are free to decide whether to continue to inflate the balloon (unless it explodes beforehand) and when to stop pumping and cash out (save) their accumulated earnings on any given trial. That is, a reward balloon trial ends when the balloon explodes or the participant decides to cash out. Participants are typically asked to earn as much money as possible completing the BART.

In a standard implementation, the BART starts off as a paradigm of ambiguity (because participants often have no representation of risk at the start of the trial), and only via repeated pumping, cash-out and explosion experiences can participants start to build up a mental representation of the “riskiness” of balloons with regards to approximate balloon inflation capacities. To manipulate riskiness, the standard implementation involves two types of reward balloon, with one featuring a higher maximum capacity (and thus higher average explosion point) than the other. Moreover, the standard implementation also yields the same reward (per pump) regardless of the level of risk (that is, regardless of how many pumps have already been administered). In the current study, we ran an adapted fMRI version of the BART, which manipulated some of the main characteristics of the standard implementation.

Specifically, we adopted a version featuring three balloon types: two different types of reward balloon (red or blue color, assignment of balloon type to color was counterbalanced across participants) and control balloons to account for motor-related activity (gray color; these did not add to participants’ earnings). For both reward and control balloons, explosion points were drawn from a uniform distribution with a minimum of one and maximum of 16 pumps (that is, the maximum capacity for reward and control balloons was 16 pumps). In contrast to standard implementations, the two types of reward balloon differed with regards to the underlying reward function. For one reward balloon type, accumulation of rewards for successful sequential pumps was driven by the standard linear payoff function, where each successful pump earns the same amount (e.g., 0.05 Swiss Francs). The second reward balloon type, in contrast, was characterized by an exponential reward function; initial reward is low and accumulates slowly, but as the balloon grows larger (i.e., the stakes rise), payment per pump increases exponentially. Simulated payment structures for both balloons are shown in Fig. A2.

Our motivation for the two types of reward balloon was twofold. First, we wanted to capture the idea that taking risks ”in the wild” may pay off in the long run, but that also means repeated exposure to and acceptance of losses. Exponential balloons simulate such a context: over time, pumping will lead to increasing rewards and higher accumulated total earnings, but in the short run require participants to explore and accept many more explosions compared with the linear balloon. A second motivation was the way in which we programmed the reward function (see Appendix A for formalization of linear and exponential reward functions), whereby exponential balloons would initially increase uncertainty because participants would experience even more ambiguity due to the initial lower reward per successful pump of exponential compared with linear balloons. We thus hoped to present participants with an experimental manipulation that would affect behavior and thereby lead to insights about behavioral and neural markers for the different reward conditions, and in particular their convergence.

Feedback was presented at the end of each trial (that is, at the point when the balloon exploded or participants cashed out) in the form of earnings for the current trial, as well as total earnings accumulated across all completed trials. We used a fixation cross to visually separate consecutive trials, and programmed intertrial intervals between trial offset and trial onset (range = 1000 − 11000 ms, mean = 4340 ms) as well as predetermined but randomly drawn interstimulus intervals between stimulus offset and stimulus onset within a trial (range = 1000 − 2000 ms, mean = 1500 ms). Performance inside the scanner was self-paced and incentivized, and earnings were paid out in cash at the end of the fMRI session.

#### Delay discounting

Delay discounting (aka temporal discounting) paradigms are commonly adopted to capture individual differences in impulsive choice (Seaman et al., 2022; Ruggeri, 2022). In a standard implementation, smaller-sooner rewards are repeatedly pitched against larger-later rewards, with trials varying with respect to the intervals of delay, reward magnitudes, and percentage differences between smaller and larger rewards. Thus, although the BART introduces uncertainty concerning balloon capacity and explosion points—the magnitude and probability of incurring rewards and losses—the delay discounting task eliminates such uncertainties. Instead, it introduces uncertainty about the future itself, including one’s own future self. Will I be around to receive the payment? Will the researchers be around to pay me? Maybe inflation will drastically change the value of the payment? These and other future-oriented questions could drive individuals’ decisions between smaller-sooner and larger-later options, making the delay discounting task a candidate measure for decision-making under a form of prospective, existential uncertainty.

In neuroimaging studies, the delay discounting task has been used to isolate and characterize the neural basis of impulsive choice (McClure et al., 2004) and also to probe age-related differences therein (Samanez-Larkin et al., 2011; van den Bos et al., 2014; Samanez-Larkin & Knutson, 2015; Eppinger et al., 2012). In this study we followed previous analyses that examined the role of the presence of immediately available rewards on both behavioral and neural indices (McClure et al., 2004) but with an additional focus on age effects (Eppinger et al., 2012).

To facilitate the desired analyses, participants completed 80 trials of the delay discounting task inside the MRI scanner. The 80 choice sets (i.e., trade-offs) were based on five unique delay-pairings, namely (a) today versus in two weeks, (b) today versus in four weeks, (c) in two weeks versus four weeks, (d) in two weeks versus six weeks, and (e) in four weeks versus six weeks. The difference between smaller and larger amounts in each choice set mapped onto eight different percentage differences (1%, 3%, 5%, 10%, 15%, 25%, 35%, 50%). We thus created 16 trials based on eight possible percentage differences for each of the five temporal pairings, amounting to 80 trials in total. The rewards in the choice set were generated by drawing 80 random numbers from a normal distribution between five and 40, and each of these 80 random values presented the smaller reward. Based on these initial values, we constructed the 80 unique choice sets by adding the required percentage differences (two trials for each of the eight possible percentage differences, for each of the five delays) to the randomly drawn numbers, which yielded the larger-later option. All participants completed the same 80 trials, but in randomized order. We programmed intertrial intervals between one and 11 seconds (mean = 4.32 s).

Performance was incentive compatible, and participants were informed at the start of the paradigm that one of their choices was to be drawn and paid out. If the selected trial included a smaller-sooner choice that was today, participants received the money at the end of the scanner session. If the drawn trial included a choice to be realized at any other time, we matched the waiting time and participants received the money in cash via registered post.

#### Covariate measures

Behavioral measures of risk preference have been shown to suffer from low convergence, at the level of both behavior (Frey et al., 2017; Frey et al., 2021; Mamerow et al., 2016) and the brain (Tisdall et al., 2020; Congdon et al., 2013). Cognitive theory [e.g., *confound hypothesis* (Olschewski et al., 2018; Mata et al., 2011)] points towards the important role of the cognitive resources required for the completion of behavioral measures, in particular when these rely on integrative processing, learning, working memory, or attention, to name but a few. As many of these cognitive processes are subject to age-related differences, the heterogeneous life span trajectories observed for risk preference measures may in part be due to these confounds rather than to changes in risk preference per se (Mata et al., 2011; Best & Charness, 2015; Mamerow et al., 2016). To account for the influence of factors that have been posited to exert a confounding influence on individual differences in decision-making under uncertainty (Frey et al., 2021), we used data collected as part of the laboratory session of the larger study (https://osf.io/uj359) to control for (socio)demographic (gender, education, income) and cognitive variables (numeracy, working memory). Participants self-reported their gender, educational attainment and monthly personal income, and both numeracy (Weller et al., 2013) and working memory (Unsworth et al., 2005) were captured via computerized tasks.

### Neuroimaging data acquisition

The neuroimaging data were collected on a Siemens 3T MAGNETOM Prisma magnetic resonance imaging (MRI) system with a 20-channel head coil at the University Hospital Basel. For every participant, we first acquired a structural T1-weighted scan using a magnetization-prepared rapid gradient echo sequence (repetition time = 2500 ms, echo time = 4.25 ms, inversion time = 1100 ms, flip angle = 7^∘^, field of view = 256 mm × 256 mm, 192 slices, voxel dimensions = 1.0 mm isotropic). This was followed by the acquisition of the task-related functional runs via T2^*^-weighted blood-oxygen-level-dependent echo-planar imaging sequences (repetition time = 2010 ms, echo time = 30 ms, flip angle = 78^∘^, field of view = 192 mm × 192 mm, voxel size = 3 mm × 3 mm × 3 mm, 33 transversal slices per volume, 15% distance factor).

### Procedure

At the time of the neuroimaging session, all participants were once again screened for any contraindications for MRI-safety, and provided with MRI-safe clothing. MRI-safe glasses were available to correct for impaired vision. Both paradigms were programmed and presented using Eprime 2.0 software, which we linked up to the scanner trigger signal to provide input for the temporal alignment of the onset of volume collection. The paradigms were presented via a projection system onto a screen behind the scanner, which participants inside the bore could see via a mirror located on the head coil. We recorded individuals’ choices using a Celeritas response system attached to their right hand. Prior to completing the two paradigms inside the scanner, participants completed short sets of practice trials to familiarize themselves with the visual and response components of both tasks.

For the BART, individuals were not told the exact reward functions underlying exponential reward balloons. Instead, participants were instructed that successful pumping on one balloon (color) would always yield the same amount, whereas the other would not, and participants would need to figure out the reward structure of this balloon themselves for optimal performance and achieving high earnings. Because exponential balloons introduce more uncertainty, they thus also afford more exploration. For the delay discounting task, participants were explicitly informed about the random selection of a trial and the realization of their choice on that particular trial, including any potential delays in receiving the reward.

To ensure (especially older) individuals were as comfortable as possible inside the scanner and for the duration of the scan session (approximately 60 minutes), we allowed for extra time at the start of the session to add cushioning and padding to individuals’ placement on the MRI table.

### Main analyses

We conducted a series of key analyses to test for age effects on behavioral and neural indices of decision-making under uncertainty. We describe each of these steps in detail below. Unless stated otherwise, all main analyses were conducted using R Studio (Core Team, 2021).

#### Behavioral analyses

##### BART

For the BART, we concatenated the two functional runs of the paradigm and analyzed individuals’ performance across the two runs. As the BART was designed to capture individual differences in risk preference (Lejuez et al., 2002), typical performance indices of risk taking comprise total number of pumps, average number of pumps, average number of pumps on trials that did not lead to an explosion (i.e., adjusted average number of pumps), and number of explosions, but also include task performance indices as captured via total earnings or reaction times (Schmitz et al., 2016). Some of the existing scoring alternatives for the BART are (highly) correlated (Mamerow et al., 2016; Tisdall et al., 2020), even though they may tap into slightly different behavioral (and potentially cognitive) components. For our main analyses of age effects, we computed the average adjusted number of pumps as this was the main BART performance indicator of interest and used it in all subsequent analyses. For descriptive purposes, we computed additional indicators (total number of pumps, number of trials, number of explosions, total earnings, reaction time), and tested the associations between different BART indices using correlation analyses. To examine if the experimental manipulation of reward function influenced behavior on the BART, we first computed one index per reward balloon type, and examined mean differences between indices for the two reward balloon types via two-sided paired-samples *t*-tests.

##### Delay discounting

Given our focus on decision-making under uncertainty, we primarily followed previous work contrasting behavioral patterns for immediate and delayed options (Eppinger et al., 2012). We computed participants’ proportion of smaller-sooner choices for trials with (a) immediate versus delayed options, (b) varying delays, and (c) varying reward differences, as well as (d) the respective reaction times. Informed by previous work finding the standard index of proportion of smaller-sooner choices to be highly positively correlated with computational indices of temporal discounting [e.g., discounting parameter *k* (Seaman et al., 2018)], we concentrated our behavioral analyses on model-free indices of delay discounting. Mirroring our BART behavioral analyses, we computed correlations between the different delay discounting metrics to ascertain their similarity.

##### Covariates

We computed one index for each of the two measures of fluid cognitive capacity. For numeracy, we computed the total number of correctly solved math problems, yielding a score between zero (no problem solved correctly) and eight (all problems solved correctly) for each participant (Weller et al., 2013). To capture individual differences in working memory from the automated operation span, we computed the total number of correctly recalled letters (recalled correctly and in the correct order) (Unsworth et al., 2005), which resulted in a score between zero (no letters recalled in the correct order) and 75 (all letters recalled in the correct order) for every participant.

#### Neuroimaging data analyses

Prior to running any analyses, we excluded neuroimaging data from 12 participants (of the recruited 189 MRI participants) due to incomplete imaging data (n = 10) or faulty equipment (n = 2). Below we describe analyses of the neuroimaging data from the remaining 177 participants. We describe the standard statistical routines implemented in SPM12 https://www.fil.ion.ucl.ac.uk/spm/software/spm12/ for all preprocessing routines of the raw functional neuroimaging (fMRI) data in the Appendix (A). After preprocessing, we excluded a further two individuals from the statistical analyses due to excessive head motion, resulting in an effective sample of 175 participants included in all subsequent analyses.

##### Individual-level contrast analyses for the BART

The BART is predicated on the assumption of being a more naturalistic paradigm then, for example, described lotteries (Schonberg et al., 2011; Rao et al., 2008; Lejuez et al., 2002; Mata et al., 2011), thus should capture the neural processes associated with pertinent risk-taking related features, including anticipated (uncertain) rewards, losses, and their integration. The structure of the BART paradigm, however, can lead to confounded processes, making it difficult to clearly isolate these different mechanisms (Schonberg et al., 2012; Schonberg et al., 2011); as pumping continues, rewards accumulate, but so does the risk of explosion and thus loss of the current reward. To deal with the paradigm’s complex (and partly confounded) structure, we sought to utilize different contrast analyses to tap into different aspects of uncertainty. Specifically, to estimate voxel-wise neural activation differences in the BART, we focused our analyses on three contrast analyses because each of these tapped into different aspects of uncertainty during decision making. For details about the underlying general linear model, see Appendix A.

##### Reward balloons versus control balloons

A standard contrast of neural activation differences in the BART is that of reward balloons versus control balloons (Schonberg et al., 2012; Rao et al., 2008; Yu et al., 2016; Tisdall et al., 2020; Wang et al., 2022). This contrast is thought to capture essential processes associated with decisions being made under uncertainty, in particular because only the balloon display phase is modeled, but not outcome or feedback phases. For the current analysis, we contrasted average activation differences (relative to baseline) of reward balloons with the average activation differences associated with control balloon trials (accounting for head motion). This contrast has been shown to yield activation differences in subcortical brain circuitry associated with the processing of anticipated (uncertain) rewards and losses, as well as with cortical integration regions, among others (Schonberg et al., 2012; Wang et al., 2022). However, although standard, this contrast misses the rising tension associated with additional pumping, because it treats every balloon equally, regardless of where in the sequence of pumps this balloon occurred.

##### Parametric modulation of reward balloon versus parametric modulation of control balloons

To capture the rising tension of additional pumping, we analyzed activation differences for the parametric modulation of the (demeaned) number of (pumps on) reward balloons and contrasted these with the parametric modulation of the (demeaned) number of (pumps on) control balloons. For this contrast, a value of zero would code for the participant’s own average number of pumps (across trials), positive values for a pump number above average, and negative values for a pump number below average. As the value of this regressor becomes more positive, the participant is exceeding their own mean pumping behavior, and is also approaching the explosion point. By better capturing the rising tension associated with each additional pump, this contrast is thought to better capture the affective component of decision-making under uncertainty. As such, the main effect for this contrast might yield parametrically increased insula activation for parametric pumps on reward relative to control balloons; as pumping goes up, so does insula activation but given the confounded nature of the paradigm (Schonberg et al., 2011), reward-related nucleus accumbens activation might also track increasing pumps.

##### Linear versus exponential reward balloons

To assess whether our experimental manipulation of reward functions affected the neural correlates of decision-making under uncertainty in the BART, we contrasted average activation differences in linear reward balloons with average activation differences in exponential reward balloons. Based on our motivation for introducing this comparison, we expected higher average activation in nucleus accumbens and insula for exponential compared with linear balloons, and potentially also higher activation in brain regions supporting learning and integrative processing.

##### Individual-level contrast analyses for the delay discounting task

To capture different facets of the temporal uncertainty experienced in the delay discounting paradigm, we focused our neuroimaging analyses of on two contrasts, one capturing *immediacy*, and another capturing *delay* (Eppinger et al., 2012; McClure et al., 2004). To avoid biasing the neural contrast analysis with an imbalanced number of trials, we removed all trials that offered a trade-off between a smaller amount in four weeks versus a larger amount in six weeks from the neural analyses (Samanez-Larkin et al., 2011). We consequently (a) worked with 32 trials involving an immediate payment and 32 trials involving delayed payments only, and (b) controlled the starting point of the delayed trials by including only trials that offered the smaller option in two weeks. Our contrast analyses thus focused primarily on the temporal characteristics (Eppinger et al., 2012; McClure et al., 2004; Samanez-Larkin et al., 2011) rather than on localizing an integrated (subjective) value signal (Seaman et al., 2018).

##### Immediacy

Following previous work (e.g., (McClure et al., 2004; Eppinger et al., 2012; Samanez-Larkin et al., 2011)), we conceptualized uncertainty in the delay discounting task as the differential processing of options with and without an immediate payoff. Specifically, we sought to contrast activation differences on trials with the sooner option being paid out *today* with activation differences on trials for which the sooner option started in two or in four weeks. Previous work suggested neural differences in the processing of immediate versus delayed rewards, including dissociation in ventral striatal and medial prefrontal cortex (McClure et al., 2004), furthermore pointing towards age-related differences in striatal reward sensitivity as driving age-related decreases in delay discounting (Eppinger et al., 2012).

##### Delay

Although uncertainty may pertain to the presence or absence of immediate options, it can also be conceptualized as the length of the delay between the sooner and the later option. To investigate the neural markers of delay and probe potential age-related differences therein, we specified a second contrast between all trials that included a two-week delay (regardless of the sooner time point) and trials that involved a longer, four-week delay. If the temporal distance between payments plays a role, we would expect activation differences for these two trial types, potentially involving higher activation in reward-related striatal regions for shorter delays. To the extent that time horizons change as a function of age (Carstensen, 2021), we may also expect that longer delays lead to age-related differences in their neural representations.

##### Volumes of interest

**Fig. 2:**
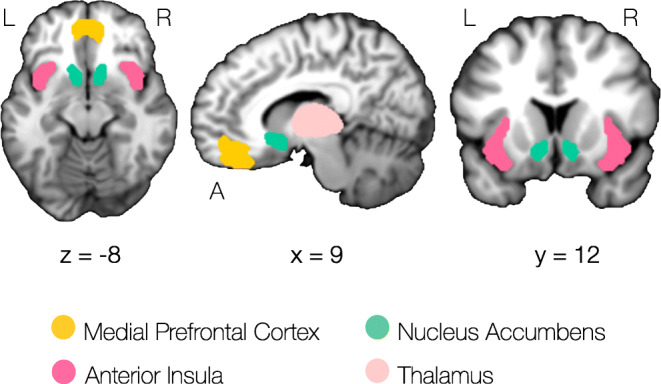
Volumes of interest used for extraction of activation differences from neural contrast analyses. Coordinates are given in MNI space

Although AIM is relevant for all aspects of the decision-making process, we were particularly interested in the first two stages; the *affective* evaluation of anticipated gains and losses, as well as *integrative* mechanisms acting on these different signals in the process of valuation. Thus, for our volume-of-interest (VOI) analyses, we focused on brain circuitry proposed to be particularly important for these initial processes, namely nucleus accumbens (processing of anticipated rewards), anterior insula (processing of anticipated losses), medial prefrontal cortex (integration of signals into subjective value) and thalamus (involved in reward learning). The VOI masks used for the current analyses are presented in Fig. 2. We provide a detailed description of mask construction in Appendix A.

##### Group-level contrast analyses

Given our focus on age-related effects on neural activation differences in a set of *a priori* theoretically derived brain regions, we only performed supplemental analyses to estimate and visualize group-level (average) contrast activation differences. Details concerning the group-level analyses and results based on 175 participants are provided in Appendices A and B, respectively.

#### Individual differences analyses

##### Bivariate associations

Our initial analyses focused on understanding bivariate associations between age, behavioral and neural markers of decision-making under uncertainty, as well as (socio)demographic and cognitive covariates. For this purpose, we first computed a matrix of Pearson correlation coefficients between all variables in our analyses. Seven individuals did not disclose their personal monthly income. In our analyses we used pairwise complete observations to compute bivariate associations, and consequently all analyses that included income were based on 168 participants, whereas analyses that did not include income were based on 175 participants. Second, we translated the correlation matrix into a network plot, visually grouping variables in order to more easily identify the extent to which different variables converge (i.e., form clusters) or diverge based on their associations to the other variables in the network. We used the R package *qgraph* (Epskamp et al., 2012) to generate the network plot, specifying a Fruchterman Reingold algorithm and a repulsion parameter of 0.7 to determine the relative spacing of variables in the network based on the strength of their associations. To focus on the most pertinent associations, we only included correlations with an absolute correlation coefficient of *r*_*Pearson*_ ≥ 0.15 in the network plot.

##### Specification curve analysis

Specification curve analysis, SCA (Simonsohn et al., 2020) provides a method to systematically assess and visualize, through exhaustive combination, the (variance in) effect sizes for a given set of predictor, outcome, and confounding variable(s). In this study, we performed one SCA to examine the effect of age on behavioral and neural indices of decision-making under uncertainty while controlling for covariates. Our SCA thus included one predictor (age), 24 outcome variables, and five covariates (sex, education, income, numeracy, working memory). The outcome variables comprised four indices of performance (three from BART, one from delay discounting), 12 neural indices from BART (three contrast analyses with extraction from four VOIs each), and eight neural indices from delay discounting (two contrast analyses with four VOIs each). The total number of specifications given the selected predictors, outcomes, and covariates was derived through additive combinations of age, outcomes, and covariates, with the restriction that a specification (i.e., a model) always contained age, one outcome, and one unique combination of covariates. This approach resulted in 24 × 2^5^ = 768 unique specifications. Every specification was estimated via ordinary least squares regression models using the R package *specr* (Masur & Scharkow, 2020). We report the median age effect across all specifications, and also report the number of null, positive, and negative age effects. For a more detailed discussion of the rationale behind SCA, see Appendix A. Due to missing income information for seven participants, specifications that included income were based on 168 participants, and specifications that did not include income were based on 175 participants.

##### Permutation testing

To ascertain the robustness of the overall set of effects, we followed a permutation-based approach (Rohrer et al., 2017) to calculate the global significance of the observed SCA. We estimated whether the empirically observed set of effects deviated systematically from the to-be-expected false-positive effects if there was, in fact, no systematic relationship between age and the outcomes of interest. For this purpose, we first generated 500 shuffled data sets by randomly sampling the age variable (with replacement), and in a second step computed a SCA for each of the 500 new shuffled data sets. We then counted how many of the shuffled new data sets yielded a larger number of age effects than observed in the original (unshuffled) data set, and divided this number by the number of shuffled data sets (i.e., 500).

## Results

### Behavioral results for BART

**Fig. 3:**
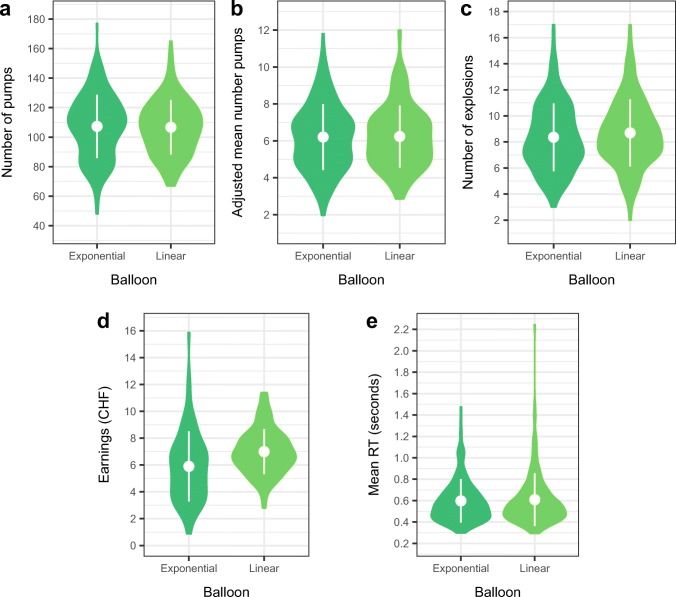
Risk taking in the BART for two types of reward balloon (N = 175). White points indicate the mean, with error bars extending to one standard deviation

In a first step, we computed summary indices of risk taking and performance in the BART, separated by reward balloon type (Fig. 3). We found no behavioral differences between linear and exponential reward balloons with regards to indices of risk taking (all *p* > 0.05) (Table B1), suggesting participants did not adjust their behavior as expected. On average, participants earned 6.45 CHF in the BART, but we did find a difference with regards to earnings in the two reward balloon types, such that participants earned significantly less money on exponential compared to linear balloons (mean difference = 1.10 CHF, *p* < 0.001). The (direction of the) difference in earnings may be due to insufficient exploration on the exponential balloon. Specifically, pumping on exponential balloons pays off in the long run (Fig. A2, right panel), but participants also need to experience relatively more explosions (i.e., losses). Thus, exponential balloons expose participants to increased uncertainty (at least initially, given the task instructions and lack of information pertaining to the exact reward structure of exponential balloons) and require more exploration. Across participants, the (adjusted) average number of pumps was much lower than what would have been necessary to experience the steep incline in payment per extra pump (Table B1), thus it is possible that participants did not fully realize the potential of exponential balloons. We visually explored age-related differences in the summary indices of risk taking and performance in the BART for the two reward balloon types (Fig. B1); the results suggest that age did not have a marked effect on BART behavioral indices. Furthermore, earnings across balloon types were similar for younger (6.40 CHF), middle-aged (6.45 CHF), and older participants (6.50 CHF).

Based on these results, we aggregated across reward balloons and performed all subsequent analyses on indices of risk taking on reward balloons. As expected, the adjusted average number of pumps was positively correlated with number of pumps (*r*_*Pearson*_ = 0.84), earnings (*r*_*Pearson*_ = 0.69) and number of explosions (*r*_*Pearson*_ = 0.44), highly negatively correlated with number of trials (*r*_*Pearson*_ = − 0.73), but not correlated with reaction time on reward balloons (*r*_*Pearson*_ = − 0.1) (Fig. B2). Based on these results, we computed an index for the adjusted average number of pumps across the two balloon types for our main analyses. However, due to the novelty of this BART implementation, in particular the idea that the exponential balloon may introduce even more uncertainty which may interact with age, in addition we also entered adjusted average number of pumps for the linear and the exponential balloon as separate outcome variables into our main analyses.

### Behavioral results for delay discounting

**Fig. 4:**
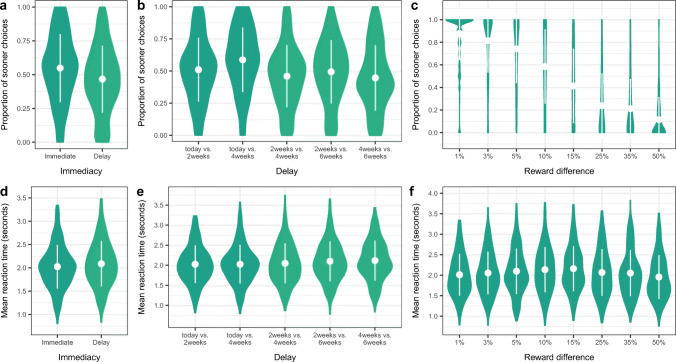
Choice proportions and reaction times in delay discounting. We plotted choice proportions as a function of *immediacy* (panel a), *delay* (panel b), and *reward difference* (panel c), and show the same plots for reaction time (panels d-f). White points indicate the mean, with error bars extending to one standard deviation

To understand general patterns of behavior for the delay discounting task in our study, we computed various choice proportions and reaction times (Fig. 4). Specifically, across the sample, we looked at the average proportion of smaller-sooner choices across all trials as well as for trials with particular properties, including *immediacy* (i.e., trials for which the smaller option was delivered today), *delay* (i.e., for the five different delays), and *reward difference* (i.e., the eight magnitude differences between the smaller and the larger option). For each of these trial types, we also analyzed mean reaction times. The overall pattern for choice proportion and reaction times mirrors previously published findings (Eppinger et al., 2012) (Table B2), including a higher mean proportion of sooner choices for trade-offs with immediate options than for sets with delayed rewards only (Fig. 4, panel a and b). Taking the smaller-sooner option was also more frequent for smaller differences between the sooner and the later options, dropping below 50% (i.e., higher proportion of delayed choices) for options with a difference of at least 15% between the smaller and the larger payment (Fig. 4, panel c). We did not see marked reaction time differences as a function of immediacy, delay, or reward difference (Fig. 4, panels c-e, Table B2 We visually explored age-related differences for the various summary indices of delay discounting (Fig. B3); the results suggest that age did not have a marked effect on delay discounting indices.

As multiple indices of choice can be used, we examined the correlation between the number and proportion of smaller-sooner choices, proportion of smaller-sooner choices when an immediate option is present, and overall reaction time. As expected, the number and proportion of smaller-sooner choices was highly correlated, yet we found no evidence for strong associations between choice and reaction time (Fig. B4). For our main analyses (and to match up with our neuroimaging contrast analyses for delay discounting), we used the proportion of immediate choices (out of all trials with a today option) in all our subsequent analyses probing age effects.

### Bivariate associations between study variables

**Fig. 5:**
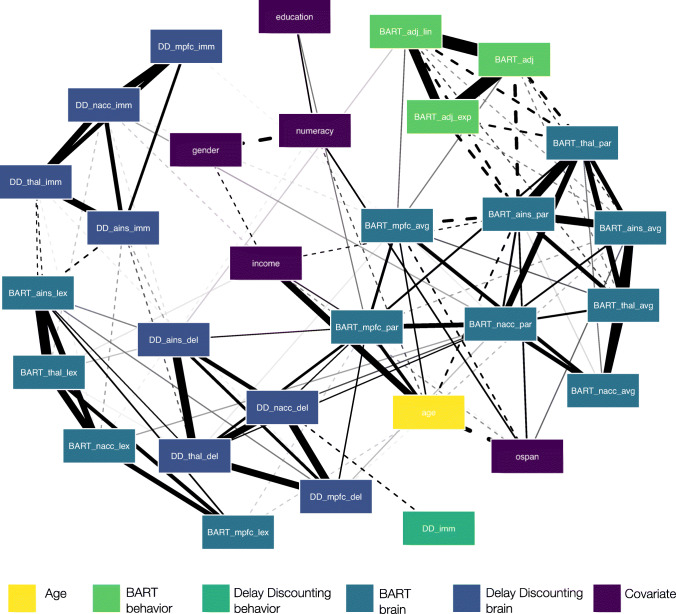
Network plot for bivariate associations between age, behavioral and neural markers of decision-making under uncertainty, and covariates. Note: Variables were visually grouped by measure. Edge thickness is indicative of the strength of Pearson correlation coefficients for associations between two variables, with solid (dotted) edges representing positive (negative) associations. We plotted correlations with absolute coefficients of *r*_*Pearson*_ ≥ 0.15

Based on the correlation matrix for associations between study variables (Fig. B6), we generated a network plot for all Pearson correlation coefficients with an absolute magnitude of *r* ≥ 0.15. As shown in Fig. 5, a few noteworthy patterns emerged. First, behavioral indices between paradigms diverged (that is, were not or negatively correlated and thus were located further away from each other in the network plot) whereas behavioral indices within paradigm (for the BART) converged (that is, were positively correlated and thus formed clusters inhabiting similar space in the network plot). Second, although behavioral markers of pumping behavior in the BART were sporadically associated with BART neural markers, the proportion of immediate choices in delay discounting was mainly uncorrelated with neural markers of delay discounting contrasts. Third, BART neural indices extracted from the average contrast of reward versus control balloons and from the parametric modulation contrast showed strong positive associations, suggesting that these two contrasts capture similar neural processes. Interestingly, this did not apply to BART indicators extracted from the contrast of linear versus exponential reward balloons, which formed a separate cluster on the opposite side of the plot. Fourth, neural markers from delay discounting correlated strongly within contrast, but were not associated across contrast. This pattern suggests that neural markers associated with *immediacy* and *delay* capture different processes.

Focusing on the main variable of interest, we observed heterogeneous associations between age and behavioral as well as neural markers of decision-making under uncertainty. For example, age was associated with neural indices extracted from the BART (primarily with indices from the average reward vs. control balloons contrast and the parametric risk modulation contrast). In contrast, age was not associated (i.e., absolute correlation coefficient lower than 0.15) with behavioral indices from either paradigm, and was also not associated with neural markers extracted from delay discounting contrast analyses. As expected, age was negatively associated with indices of cognitive capacity and positively associated with income.

To ascertain the robustness of the observed associations for age, in particular when controlling for covariates, we adopted a SCA (Simonsohn et al., 2020), a multiverse analytic approach to test associations between age and markers of decision-making under uncertainty for various combinations of covariates.

### Specification curve analysis

**Fig. 6:**
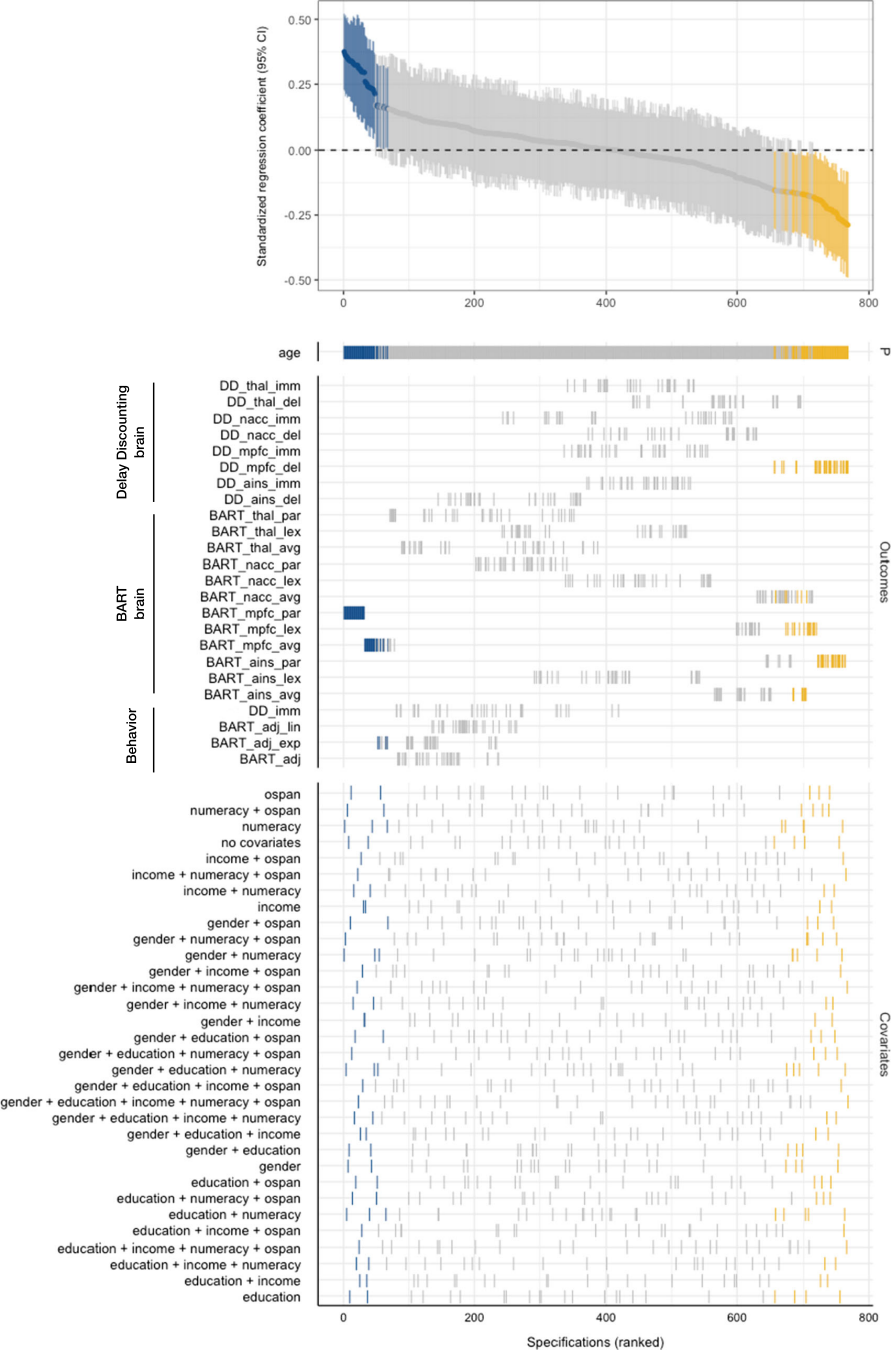
Specification curve analysis. Top panel displays the standardized regression coefficients (95% CI) for age on varying outcomes of decision-making under uncertainty, ordered by effect magnitude. Tick marks in the lower panels indicate the exact specifications. P = predictor. Colors indicate significant (p ≤ 0.05) positive (blue) and negative (orange) effects

A summary of the number of empirically observed age effects is provided in Table 1. As shown in the top panel of the specification curve (Fig. 6), we obtained mainly null effects of age on varying operationalizations of uncertainty, both for behavioral and neural markers (n null effects = 624). This conclusion is also reflected in the median age effect across all 768 unique specifications being 0.008 (i.e., close to zero). Looking at particular sets of specifications, we found no significant associations (at *p* = 0.05) between age and delay discounting; older adults did not take the immediate option less often than younger participants, nor did we find evidence for neural activation differences in the four regions of interest for the *immediacy* contrast. We did see an effect of age on Medial Prefrontal Cortex (MPFC) activation differences for the *delay* contrast (median adjusted *R*^2^ = 0.03 for 32 significant specifications), suggesting that the difference in MPFC activation for delays of two weeks versus four weeks becomes smaller with increasing age. Interestingly, we found no effect of age on nucleus accumbens activation differences, suggestive of preserved reward sensitivity during delay discounting (Eppinger et al., 2012). The latter results may also explain the absence of any behavioral effects of age observed in this study and in previous meta-analytic work (Seaman et al., 2022).

**Table 1:** Summary of age effects estimated using specification curve analysis

Number of specifications	768 (100%)
Number of positive effects	58 (7.55%)
Number of negative effects	86 (11.20%)
Number of null effects	624 (81.25%)
Median age effect	0.008
Number of shuffled data sets with > 144 significant specifications	7/500
Permutation test *p* value	0.014

The results for age effects on BART indices were more heterogeneous for both behavioral and neural markers. First, although we observed positive age effects for four (12.5%) unique specifications that included the adjusted number of pumps for exponential balloons as an outcome variable (Fig. 6), we found no robust associations between age and behavioral BART indices at the level of bivariate associations (Fig. 5). This divergence of results suggests that the specificity and robustness of this association remains to be clarified. Second, we observed some convergence of age effects on neural indices extracted from the BART. Specifically, age was negatively associated with insula activation extracted from the contrast of reward and control balloons (median adjusted *R*^2^ = 0.02 for eight significant specifications) and the contrast between parametric modulation of risk in reward versus control balloons (median adjusted *R*^2^ = 0.07 for 24 significant specifications). Furthermore, age correlated positively with MPFC activation extracted from the contrast of reward and control balloons (median adjusted *R*^2^ = 0.05 for 22 significant specifications) and MPFC activation extracted from the contrast of parametric modulation of risk in reward versus control balloons (median adjusted *R*^2^ = 0.12 for 32 significant specifications). Age was also found to be negatively associated with nucleus accumbens activation differences extracted from the reward versus control balloons contrast (median adjusted *R*^2^ = 0.01 for six significant specifications), and positively associated with MPFC activation extracted from the contrast of linear versus exponential balloons (median adjusted *R*^2^ = 0.01 for 16 significant specifications). Regardless of contrast, we found no associations between age and thalamus activation differences.

Overall, the SCA approach allowed us to systematically and exhaustively analyze age effects on varying operationalizations of decision-making under uncertainty, not only confirming the bivariate analyses, but also yielding a clearer, visually more accessible idea of the convergence and divergence of age effects given different outcome variables and (combinations of) covariates. In summary, operationalization—that is, how we assess individual differences in uncertainty-based decisions—seems to play a potentially larger role than the inclusion or exclusion of different covariates.

### Permutation testing

To assess the global significance of the SCA, we adopted a permutation-based approach to generate a distribution of false-positive results under the null hypothesis (Fig. B7). Out of 500 shuffled data sets (that is, data sets for which we randomly sampled the age variable with replacement), seven yielded a higher number of significant effects than observed in the unshuffled data (number of significant effects in the unshuffled data = 144). In other words, if there were in fact no systematic association between age and the outcome variables, the probability of observing the global set of significant specifications as observed here would be 1.4% (Table 1).

## Discussion

In this study we aimed to systematically examine age effects on different behavioral and neural indices of decision-making under uncertainty in a large sample of participants between 16 and 81 years of age (N = 175) while controlling for theoretically relevant covariates, including gender, education, income, and cognitive ability. We used two common decision-making tasks, the BART and a delay discounting task, as these are thought to capture decisions under uncertainty in the context of risk and trading off temporal options, respectively. In addition to adopting different tasks, we also computed different neuroimaging contrasts as these allowed us to examine whether age differentially affects the contrast-specific neural processes (e.g., immediacy vs. delay of temporal options). In what follows, we summarize our results in the context of previous findings, discuss pertinent implications of our findings, and highlight some limitations while providing an outlook on how to advance our understanding of the effects of age on dealing with uncertainty.

### Summary of results and implications

Overall, we found no evidence for wide-ranging age effects on decision-making under uncertainty. First, with respect to behavior, we found no robust age effects on risk-taking behavior in the BART, nor did we find evidence for age impacting trade-offs between present and future rewards. Thus, although some have suggested behavioral differences between younger and older adults (Wilson et al., 2021; Henninger et al., 2010; Eppinger et al., 2012), our results are in line with studies reporting no effect of age on either the BART or the delay discounting task (Yu et al., 2016; Seaman et al., 2022). Second, with respect to neural correlates, our results were more heterogeneous, mainly as a function of the two different paradigms.

For the BART, we observed negative effects of age on insula activation for both the average and parametric activation contrast, and both of these contrasts also yielded positive effects of age on activation differences in medial prefrontal cortex. Concerning the former, the observed age-related reductions in anterior insula activation are in line with previous findings (Samanez-Larkin & Knutson, 2015), suggesting that the dampened neural coding of anticipated (that is, uncertain) losses presents a key mechanism for explaining why older adults may display more risk-taking behavior than younger adults (Cavanagh et al., 2012). However, as we found no effect of age on behavior, and behavior was overall not related to neural markers, the observed age effects on anterior insula activation appear to be inconsequential for behavior in the BART. Interestingly, the observed positive effect of age on medial prefrontal cortex activation corroborates our previous finding of decreased deactivation in older relative to younger adults in the BART (Yu et al., 2016). Overall, the observed age effects on medial prefrontal cortex activation in the BART are in line with previous results, suggestive of age differences in the integration of core components such as anticipated rewards and losses into a subjective value signal (Samanez-Larkin et al., 2011; Samanez-Larkin & Knutson, 2015). In contrast, recent work using simple decision scenarios (e.g., delay discounting) found no effect of age on neural subjective value (Seaman et al., 2018), yet the presence of more complex, to-be-integrated decision components in paradigms like the BART may account for the different results (Olschewski et al., 2018; Mata et al., 2011; Seaman et al., 2018).

Concerning contrast selection, we only found negative effects of age on nucleus accumbens activation for the average but not the parametric contrast analysis of reward versus control balloons. Mechanistically, this pattern of age effects on nucleus accumbens activation has been suggested to be indicative of older adults experiencing more difficulty with the representation of a clear reward prediction error signal due to suboptimal reward learning (Samanez-Larkin & Knutson, 2015). This explanation may, in fact, account for the divergence of age effects on nucleus accumbens activation as a function of contrast, because the parametric contrast aims to identify regions which track progress over time, in particular the escalating risk associated with additional pumps, yet this should in principle be uncorrelated to reward prediction error. However, as this is the first time that age effects have been systematically studied on different BART contrast analyses, the robustness of this finding needs to be examined. Furthermore, we found that age was negatively associated with medial prefrontal cortex activation for a novel contrast of linear versus exponential reward balloons, a pattern which yet again points towards age-related differences in the computation of an integrated value signal (Samanez-Larkin & Knutson, 2015). We should note that we did not find robust behavioral differences between the two reward balloons, thus it is currently unclear to what extent the observed age effect in medial prefrontal cortex for this particular contrast can illuminate age differences in decision-making under uncertainty.

For the delay discounting paradigm, we only observed an effect of age on medial prefrontal cortex activation for shorter relative to longer delays, but did not find the previously reported age effects on ventral striatal activation differences observed for immediate versus delayed options (Eppinger et al., 2012; Samanez-Larkin et al., 2011). Thus, our results are suggestive of preserved reward sensitivity when trading off smaller-sooner versus larger-later options. Similar results were reported by Seaman and colleagues (Seaman et al., 2018), who directly targeted age differences in the neural representation of subjective value in varying discounting paradigms, also finding no age differences. More generally, the lack of age differences in the delay discounting paradigm poses questions for the mechanistic role that changing time horizons have been proposed to play across the adult life span (Carstensen, 2021); we return to this point in discussing the limitations of our work.

In the aggregate, our results suggest some age differences in the processing of anticipated gains and losses in the context of the BART but these effects do not generalize to the delay discounting task, thus limiting support for the idea of general age-related differences in the processing of uncertainty.

Our results also have some important methodological implications. First, our results suggest that the choice of task matters for identifying age differences in the neural basis of decision-making under uncertainty. We considered only two tasks but a research agenda that aims to broadly cover the links between aging and uncertainty would require examining many more different measures that have so far coexisted in the literature but only rarely been compared directly in within-subject designs. We hope to see future work covering multiple tasks and task variations that can provide a better understanding of the task characteristics, if any, that elicit age differences when dealing with uncertainty. Second, our results suggest that there is some, but limited, contribution of the choice of neural contrasts (e.g., average vs. parametric) in identifying age differences in the neural basis of decisions under uncertainty. This is important because it suggests that heterogeneous results found in the past or future literature may not principally derive exclusively from the choice of contrast. That said, it would be interesting to assess to what extent this conclusion is specific to the tasks we considered or whether this is a more general characteristic of the decision paradigms currently used in the literature. Third, we found that the inclusion of covariates in our analyses, such as measures of cognitive ability, did not play a major role in determining individual and age differences observed, which is against the idea that cognitive ability is a major determinant of age differences in tasks, such as the BART, that rely on updating beliefs over time (Mata et al., 2011). Future work needs to determine if this hypothesis needs to be rejected completely, or, alternatively, what the boundary conditions are for cognitive task demands to exact an influence on age effects on decision-making under uncertainty. Furthermore, although our paradigms were incentive-compatible and paid out real financial rewards, both previous research (Horn & Freund, 2022) and our results for specifications that included income suggest that certain methodological choices—like the incentive structure or controlling for individuals’ ability to “buffer” financial losses—may not be as potent a determinant of decision-making under uncertainty as previously assumed (Camerer & Hogarth, 1999).

### Limitations and future directions

There are five main limitations to our work that we should point out. First, we should note limitations concerning our choice of tasks for investigating age differences in dealing with uncertainty. Our work focused on two very different tasks that were not specifically designed to isolate specific mechanisms of uncertainty in a comparable fashion across the two. Further, although some have praised measures, like the BART, that simulate the thrill and engagement of real-world risk taking, others have criticized the difficulties in isolating specific cognitive and neural processes in such paradigms (Schonberg et al., 2011) and non-representative task design (Steiner & Frey, 2021). More broadly, recent work has pointed out some limitations of behavioral paradigms concerning, for example, their test-retest reliability (Frey et al., 2017) that pose a challenge for individual and age differences research. Concerning the delay discounting task, we focused on a small set of magnitudes and time intervals, which can also limit the conclusions for age differences that perhaps are expressed more clearly at longer time intervals (Seaman et al., 2022; Leverett & Garza, 2022). Moreover, while we focused our analyses on age effects observed via temporal contrasts—as opposed to, for example, on tracking subjective value across the brain (Seaman et al., 2018)—examining the neural conjunction of temporal contrasts and subjective value can shed more light on the common and unique computations captured by the different analytical methods. Future work could benefit from expanding the battery of measures as well as from conducting more thorough measure development for the purpose of neuroimaging research in order to adopt measures that can isolate particular processes and that have the desired psychometric properties.

Second, we introduced a novel experimental manipulation concerning the use of exponential and linear reward functions in the BART. The rationale for investigating different reward functions was that this could allow better discrimination of the role of exploration and varying values of uncertainty. Although we did not collect feedback from participants about whether they detected a difference between balloon types, we did not observe noticeable behavioral or neural differences between the two balloon types, suggesting that participants may not have perceived them as sufficiently different or did not have enough opportunity to learn about these differences in our study. We believe that future studies should nevertheless consider more extensive testing of different formulations of such principles, for example, in representative task designs that more strongly vary learning opportunities or measure participants’ beliefs over time (Steiner & Frey, 2021) to help uncover individual and age differences in dealing with uncertainty.

Third, recent work has called for increased attention to issues of sample size and power in neuroimaging studies (Marek et al., 2022). We have aimed to address some of these concerns by targeting neural regions of interest derived from the aging literature and adopting a multiverse approach to provide protection concerning spurious findings. In the future, however, similar work may profit from using larger samples stemming from extant panels (e.g., Dunedin Study, Lifebrain) or establishing research consortia that can more easily target larger samples for discovery purposes of neural markers of age differences in decision-making under uncertainty.

Fourth, we focused on a limited set of functional activations from a small set of brain regions and thus our results cannot provide a full account of the computational role of these regions or their role in a larger network of regions. Past work suggests that functional and structural connectivity between key neural regions can account for some individual (age) differences in decision making (Samanez-Larkin et al., 2011; van den Bos et al., 2014; Kohno et al., 2017; Leong et al., 2016) and future efforts could use some of the data reported here or adopt similar approaches to assess the role of brain connectivity in accounting for age differences in decisions under uncertainty. However, our decision to focus *a priori* on a small set of well-described regions with empirically demonstrated relevance for age effects on decision making (Samanez-Larkin & Knutson, 2015) was motivated by the desire to follow a principled approach to the selection of outcome variables; less principled approaches (Marek et al., 2022) have often resulted in the (unsurprising) convergence of effect sizes around zero, thereby masking and, potentially, undermining the contribution of neuroimaging approaches to our understanding of the role of biological markers to complex phenotypes.

Fifth, and finally, we focused our efforts on understanding the relatively well-controlled domain of the laboratory but cannot provide insight into the consequences of age differences in the neural basis of decision-making under uncertainty for real-world, everyday decisions. Future efforts could consider adopting a more ambitious strategy that integrates laboratory testing with a battery of measures covering more consequential decisions and real-world outcomes, such as financial status or health choices (Li et al., 2015); only then will we be able to make a contribution to determining the role of neural aging in how people treat uncertain gains and losses “in the wild”.

### Conclusion

We have examined adult age differences in decisions under risk using two behavioral tasks. Our results provide little evidence for behavioral differences but point out a few age-related differences in the processing of anticipated gains and losses that may be associated with uncertainty but are evident in only one of the tasks. As a consequence, our work suggests the need for a larger research agenda that considers how different forms of uncertainty and associated task characteristics determine age differences in decision making.

## Electronic supplementary material

Below is the link to the electronic supplementary material.

(PDF 4.00 MB)

## Declarations

### **Additional information**

This work is accompanied by supplemental materials, Appendices A and B.
