# Supplementary material for: Age differences in the neural basis of decision-making under uncertainty
Source: Cogn Affect Behav Neurosci. 2023 Mar 8;23(3):788–808. doi: 10.3758/s13415-022-01060-6 (PMC10390623; doi:10.3758/s13415-022-01060-6)
Supplement: Supplementary file 1 — (PDF 4.00 MB) [file 13415_2022_1060_MOESM1_ESM.pdf]

Appendix A

SUPPLEMENTAL METHODS

Power analyses and sample characteristics

For the larger research study we recruited a convenience sample of 200 healthy, right-handed adult human volunteers between 16 and 81 years of age. The original sample size was informed by power analyses in G*Power using standard methods under the assumption of using linear regression models to estimate age effects on the primary outcomes of interest. A sample larger than 80 participants was determined to yield 80% power to detect medium effect sizes in a cross-sectional analyses. The comparatively larger recruited sample reflects our interest in investigating individual differences, as well as our initial intention to extend the study to a longitudinal design; both components require a sufficiently large (initial) sample to achieve and retain sufficient power. Simulation methods assuming the use of bivariate latent growth curve models suggested 200 individuals to be sufficient to detect medium size effects with 80% probability in longitudinal analyses.

To ensure participants' safety during the neuroimaging session, we conducted a thorough screening for any contraindications to MRI safety, and excluded individuals if they reported having permanent implants (e.g. pacemaker, cochlear implant, neurostimulator, insulin pump), claustrophobia, tinnitus, epilepsy, permanent metal in or on the body (e.g., surgical clip, metal splinter, metal prosthesis, copper coil, artificial heart valve), had previously had heart or brain surgery, reported suffering from any conditions which prevented them from lying (comfortably) still inside the scanner, were pregnant, or reported the use of prescribed medication which could interfere with cognitive and neural function.

Figure A1 summarizes the study's effective sample (N=175) with regards to its (socio)demographic characteristics.

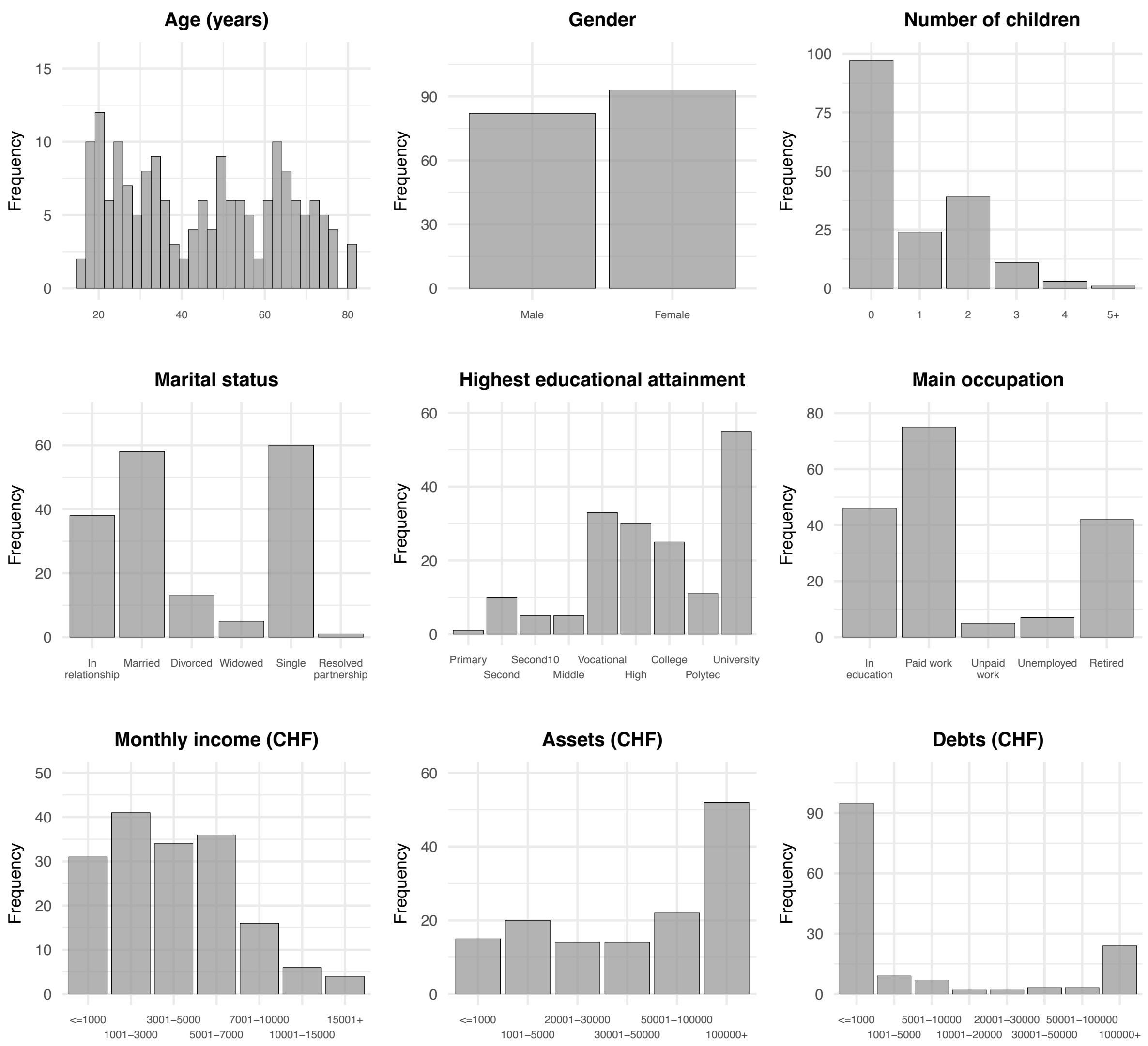

Figure A1

Demographic and sociodemographic characteristics of the study sample (N=175).

BART reward functions

In this study we introduced two balloons with the same maximum capacity but for which the accumulation of reward was driven either by a linear or an exponential reward function (Figure A2). We formalized the difference between the linear and exponential reward balloons as follows:

$$R_{lin} = p * x \tag{A1}$$

where p = reward for each successful pump (i.e., 0.05) and x = 1:number of maximum pumps (i.e., 16). The other balloon type, in contrast, was based on an exponential

reward function of the form

$$R_{exp} = x^{2.1}/100 \tag{A2}$$

where x = 1:number of maximum pumps (i.e., 16). For both balloons, the conditional explosion probability (Figure A2, middle panel) was calculated as

$$P = 1/(n - i + 1) \tag{A3}$$

where n = maximum number of pumps and i = current pump. Consider a balloon with a maximum capacity of 16 pumps (i.e., the overall maximum capacity). The respective explosion probabilities are: 0.062 (first pump), 0.067 (second pump), 0.071 (third pump), 0.077 (fourth pump), 0.083 (fifth pump), 0.091 (sixth pump), 0.100 (seventh pump), 0.111 (eight pump), 0.125 (ninth pump), 0.143 (tenth pump), 0.167 (11th pump), 0.200 (12th pump), 0.250 (13th pump), 0.333 (14th pump), 0.500 (15th pump), and 1.000 (16th pump). To get a better intuition about the exponential function describing these explosion probabilities, it is informative to start at the end: given all 15 previous pumps were successful and did not lead to an explosion, the balloon will definitely explode after the 16th pump (probability = 100%). In contrast, given all 14 previous pumps were successful and did not cause an explosion, the 15th pump has an explosion probability of 50% because the 15th pump (for a balloon with a maximum capacity of 16) can either explode and finish the trial, or not explode and result in the possibility for one last pump. Based on the conditional explosion probability, we computed the Expected Value (Figure A2, right panel) for a given pump and balloon type as:

$$EV_{lin} = \Pi(1 - P) * R_{lin} * ntrials \tag{A4}$$

$$EV_{exp} = \Pi(1 - P) * R_{exp} * ntrials \tag{A5}$$

where ntrials = 20 (the average number of trials per balloon type completed in previous studies with similar length and maximum pumps).

Preprocessing of neuroimaging data

We processed the raw images from from the BART and the delay discounting task in the same way. First, we used a two pass procedure to spatially realign

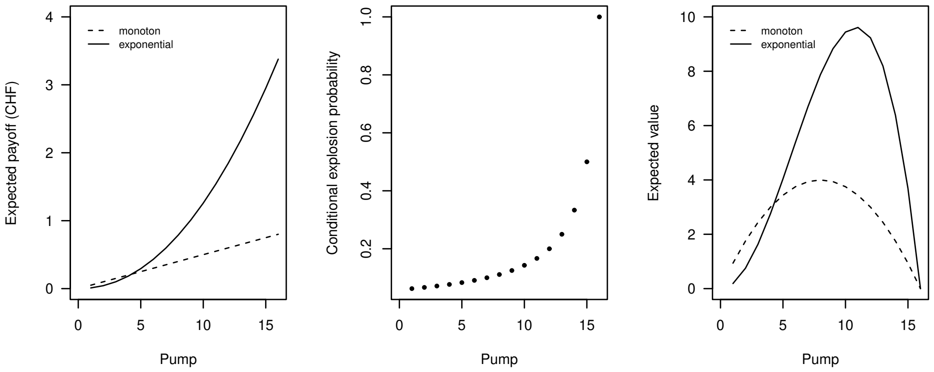

Figure A2

Risk and reward in the fMRI version of the BART.

participants' functional volumes to the series' mean image, saving out realignment parameters for six directions (three parameters for translation, three parameters for rotation). Second, to account for the interleaved (bottom-to-top) acquisition of the functional volumes, we implemented a standard SPM12 slice time correction routine. Third, we coregistered the spatially and temporally realigned functional volumes to the structural (T1-weighted) volume via maximization of a normalized mutual information objective function. Fourth, we segmented participants' structural volumes, and applied the information gained from the segmentation routine to the normalization of the functional volumes from native to MNI space via individuals' structural volumes. Fifth, we smoothed the realigned, coregistered and normalized functional volumes using a 4 mm full-width half-maximum Gaussian kernel (Sacchet & Knutson, 2013). To account for the heterogeneity of the current sample with regards to age and the impact this may have on automated preprocessing routines, we manually inspected all functional volumes of all participants, but found no evidence for suboptimal or failed normalization or misalignment. However, inspection of the realignment parameters saved out for each functional run identified two participants with excessive head motion, defined here as motion with >4 mm absolute volume-to-volume translational differences; these were excluded from all further analyses. The effective sample thus included 175 participants.

Individual-level contrast analyses for the BART

All contrast analyses were set up in the same general linear model by concatenating over the two BART runs. The structure of the BART allows for different ways of aggregation, e.g., over trials (i.e., over a sequence of balloon displays until the participant decides to cash out or the balloon explodes) or displays (i.e., individual displays and decisions within a trial). Looking at the top row in Figure 1, for a trial, we would aggregate and model activation over the entire row of events (e.g., across the first four balloons), whereas for a display, we would aggregate and model activation separately for each of the first four balloon display appearances. We specified a variable epoch model to capture neural activation over the course of one balloon display (that is, from balloon display onset to balloon display offset once a decision to pump or cash-out was recorded). We modeled each balloon display as a boxcar function, with the length being equal to the participant's balloon-specific reaction time (i.e., the time from display onset until a choice was recorded). In the general linear model, we included separate onset vectors for linear, exponential and control balloons (i.e., displays), as well as parametric modulation regressors for each of these balloon types, reflecting the demeaned pump number with regard to the entire trial. To explicitly model and thus remove other trial-relevant events from the implicit baseline activation, we also included onset vectors for explosions, as well as the six head motion parameters estimated during the spatial realignment procedure. Although the completion of the BART was self-paced, all participants completed a sufficient number of linear (mean=20.50, median=20, range=15-25), exponential (mean=20.45, median=20, range=14-26), and control (mean=20.59, median=21, range=15-27) balloon trials for analysis.

Individual-level contrast analyses for the delay discounting task

Mirroring contrast analyses for the BART, we specified one general linear model for all analyses, and used boxcar functions with individuals' trial-specific reaction time to model activation differences in the delay discounting task, from display (trial) onset to display (trial) offset. For delay discounting, we thus define a trial as the period from the onset of the display presenting a choice between a smaller-sooner and a larger-later

option to the offset of the display once a choice is recorded (or five seconds have elapsed without a response being recorded). To specifically capture neural activation associated with the "temporal uncertainty" aspect of choices between smaller sooner and larger later rewards, we sorted and contrasted (a) trials with and without an immediate option, and (b) trials with shorter (two weeks) and longer (four weeks) delays. In other words, we did not focus our analyses on capturing age differences in the neural representation of the subjective value of choice options, or, put differently, in identifying age effects on activation differences tracking subjective value (cf. Seaman et al., 2018).

Volumes of Interest

In addition to selecting VOIs based on theoretical accounts of their involvement with particular pertinent decision processes and their potential role for driving age effects therein (Samanez-Larkin & Knutson, 2015), we defined all VOIs structurally; that is, based on anatomical boundaries defined in the Harvard-Oxford probabilistic cortical and subcortical structural atlases (Desikan et al., 2006) to further circumvent arguments of circularity (Vul & Pashler, 2012). To create the binary VOI masks, we first saved out masks for "Frontal Medial Cortex", "Left Accumbens", "Right Accumbens", "Insular Cortex", "Left Thalamus" and "Right Thalamus" (as per atlas labels). All masks were then thresholded at 20 to exclude voxels with a less than 20% chance of belonging to the particular region of interest. As we had no hypothesis regarding laterality of age effects, we combined the masks for left and right nucleus accumbens and thalamus. Given the theoretical focus on the anterior insular cortex (Samanez-Larkin & Knutson, 2015), we further cut the insular cortex mask at $y = 0$ in order to exclude more posterior insula regions. All masks were binarized and used for the extraction of mean activation differences computed in our individual-level contrast analyses (Figure 2).

Group-level contrast analyses of brain activation differences

Group-level activation maps are one way to visually display average activation differences; that is, to show in which brain regions, on average, activation differences can be observed for a given statistical (contrast) analysis. In this study we did not focus

on group-level activation maps because we were interested in individual differences, which may, in fact, be masked by group-level activation differences. However, to place our neuroimaging contrast analyses into the context of previous research using the same behavioral paradigms and (similar) contrast analyses, we computed one-sample t-tests to examine whether voxel-wise activation differences obtained from contrasting different trials and time periods in the two behavioral paradigms differed significantly from zero at the level of the group. We used the Multi-image Analysis GUI MNI sample image (https://ric.uthscsa.edu/mango/mango.html) to visualize group-level maps of contrast activation differences. Anatomical labels were taken from the Neuromorphometrics Atlas in SPM12. Coordinates (in mm) are reported in standard Montreal Neurological Institute (MNI) space.

Specification Curve Analysis

SCA has previously been used in different contexts to address a variety of research questions (e.g., Frey et al., 2021; Lejarraga et al., 2019; Orben & Przybylski, 2019; Rohrer et al., 2017). In the current study, SCA allowed us to estimate the effect of age on different behavioral and neural indices of decision-making under uncertainty, while controlling for varying combinations of covariates. By "packaging" all relevant and reasonable analytic specifications (i.e., unique models) with age as a predictor, we aimed to perform a principled yet exhaustive quantification of the effect of age on different outcomes. For example, we opted to operationalize uncertainty with regards to two behavioral paradigms capturing different aspects of uncertainty, and for each paradigm we computed both behavioral and neural indices. We further distinguish between different behavioral indices, as well as between different contrast analyses of brain activation in response to the two paradigms, and between different theoretically justified brain structures (Samanez-Larkin & Knutson, 2015). Our main motivation behind performing a SCA was thus to increase methodological and outcome transparency via a multiverse approach (Steegen et al., 2016) to data analysis, allowing us to speak to the convergence or divergence of age effects as a function of the measures we chose to capture individual differences in decision-making under uncertainty.

Appendix B

SUPPLEMENTAL RESULTS

Behavior in the BART

Table B1

Risk taking in the BART for two types of reward balloon (N=175).

Index	Linear balloons	Exponential balloons	
	M (SD)	M (SD)	*t(df)*
Adjusted average pumps	6.24 (1.69)	6.21 (1.79)	0.34 (174)
Total number of pumps	106.78 (18.46)	107.30 (21.42)	-0.29 (174)
Number of trials	20.50 (2.15)	20.45 (2.27)	0.72 (174)
Number of explosions	8.70 (2.58)	8.36 (2.60)	1.47 (174)
Total earnings (CHF)	7.00 (1.68)	5.90 (2.61)	4.43 (174)***
Reaction time (seconds)	0.61 (0.25)	0.60 (0.20)	1.19 (174)

Note. ***p<0.001.

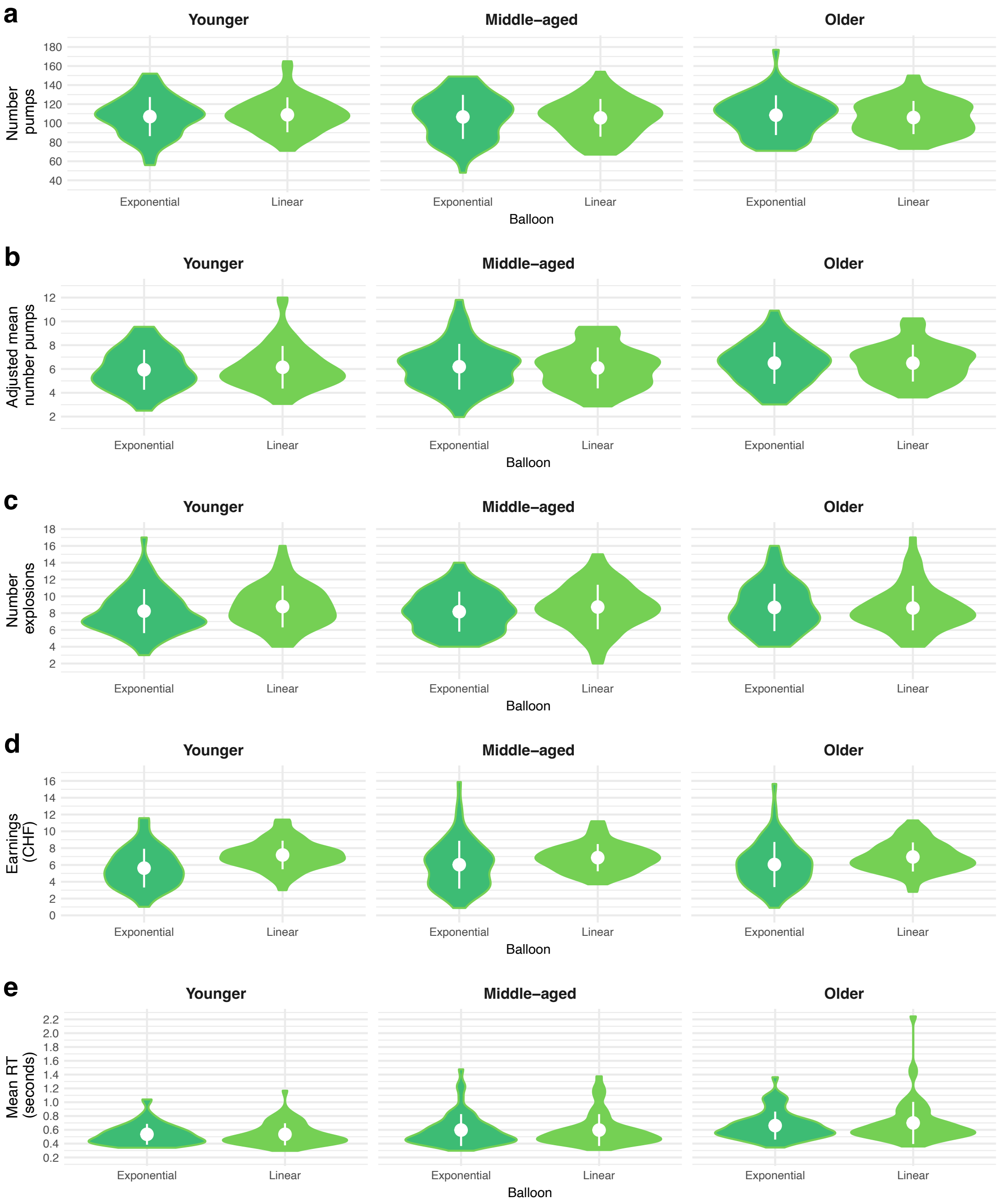

Figure B1

Age-related differences in BART performance, organized by reward balloon type. Younger = 16.1-32.09 years (n=59), Middle-aged = 32.1-55.49 years (n=58), Older = 55.5-81.4 years (n=58). White points indicate the mean, with error bars extending to one standard deviation.

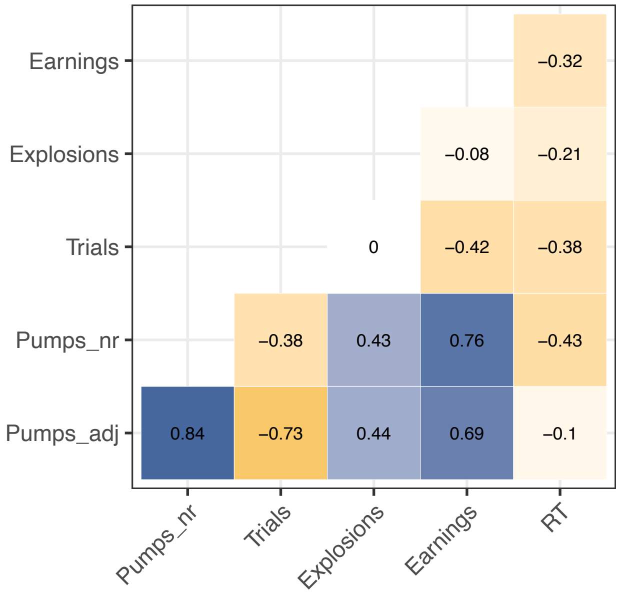

Figure B2

Correlation between indices of risk taking and performance in the BART. Note: Pumps_nr=total number of pumps on reward balloons; Pumps_adj=adjusted average number of pumps on reward balloons; RT=average reaction time for reward balloons.

Behavior in Delay Discounting

We performed descriptive and inferential analyses to examine behavior in the delay discounting task, in particular as a function of whether an immediate choice was presented (*Immediacy*), the delay between the sooner and the later option (*Delay*), as well as the difference between the smaller and the larger option (*Reward difference*). The statistics reported in Table B2 correspond to Figure 4.

Table B2

Behavior in the delay discounting task (N=175).

	Choice proportion (S-S)	RT (sec)
Trial type	M (SD)	M (SD)
All trials	0.50 (0.23)	2.06 (0.47)
Immediacy		
Immediate option present	0.55 (0.25)	2.02 (0.47)
Only delayed options	0.47 (0.25)	2.09 (0.49)
Delay		
Today versus 2 weeks	0.51 (0.25)	2.02 (0.47)
Today versus 4 weeks	0.59 (0.25)	2.02 (0.48)
2 weeks versus 4 weeks	0.46 (0.24)	2.05 (0.50)
2 weeks versus 6 weeks	0.50 (0.25)	2.10 (0.50)
4 weeks versus 6 weeks	0.45 (0.26)	2.12 (0.50)
Reward difference		
1%	0.88 (0.23)	2.01 (0.51)
3%	0.81 (0.28)	2.05 (0.52)
5%	0.74 (0.30)	2.10 (0.56)
10%	0.59 (0.33)	2.13 (0.55)
15%	0.41 (0.33)	2.16 (0.55)
25%	0.24 (0.29)	2.07 (0.57)
35%	0.21 (0.26)	2.05 (0.56)
50%	0.12 (0.20)	1.95 (0.54)

Note. S-S = Smaller-sooner; RT = reaction time; M = mean; SD = standard deviation.

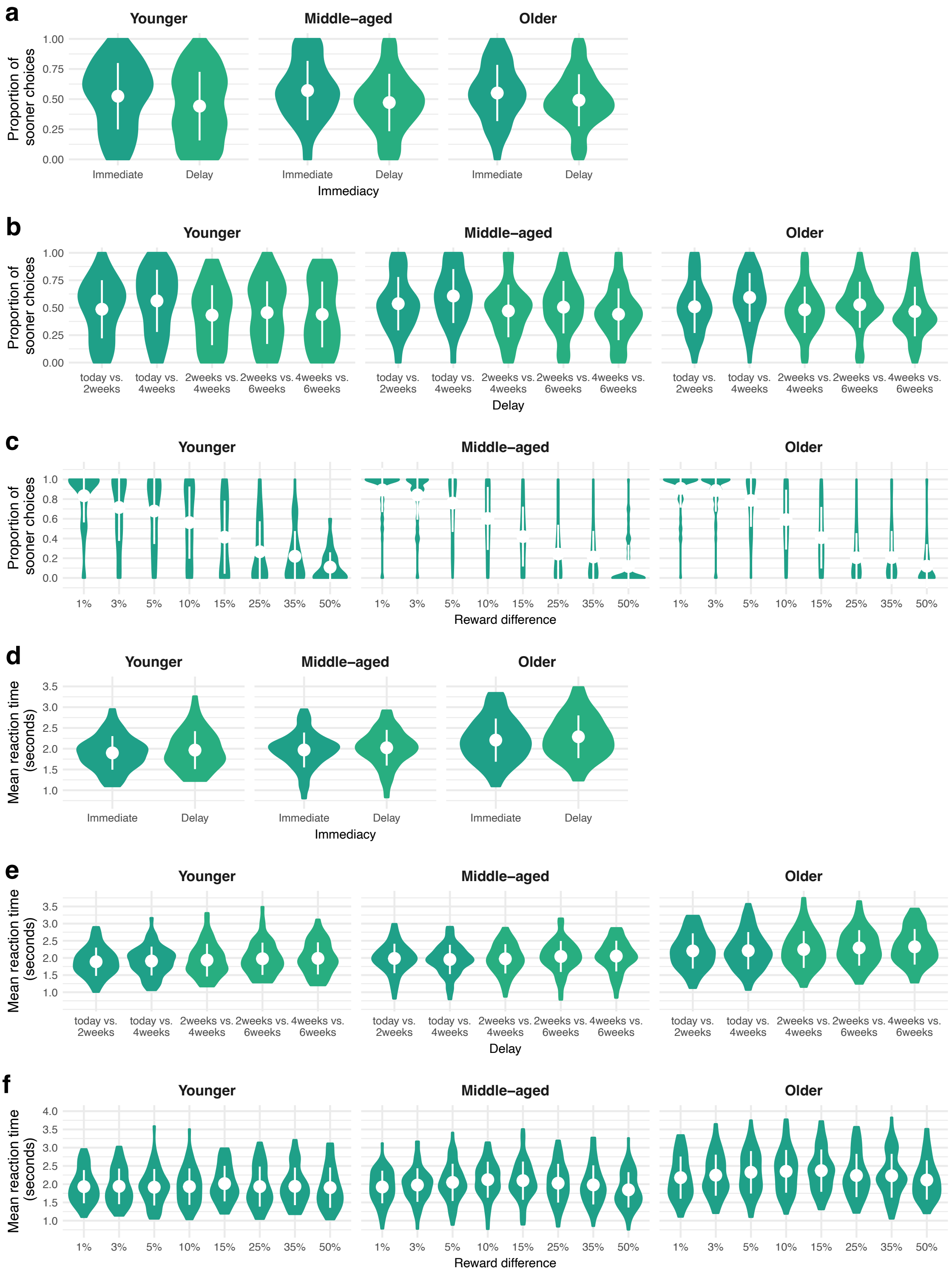

Figure B3

Age-related differences in the delay discounting task. Younger = 16.1-32.09 years (n=59), Middle-aged = 32.1-55.49 years (n=58), Older = 55.5-81.4 years (n=58). White points indicate the mean, with error bars extending to one standard deviation.

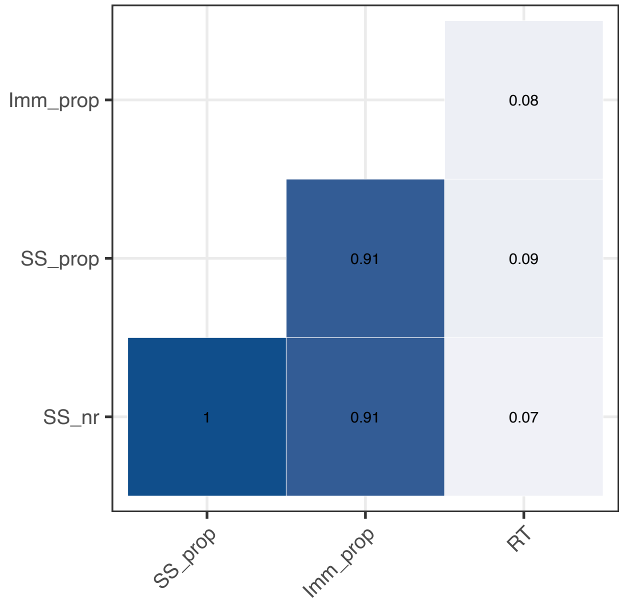

Figure B4

Correlation between indices of delay discounting. Note: SS_nr=number of smaller-sooner choices; SS_prop=proportion of smaller-sooner choices; Imm_prop=proportion of 'today' choice on trials with an immediate option; RT=mean reaction time across all trials.

Group-level maps of activation differences associated with decision-making under uncertainty

Group-level whole brain activation differences for BART and delay discounting are shown in Figure B5. These group-level analyses did not inform our individual differences analyses because we relied on theoretically defined VOIs instead of peak activation differences observed as a result of the contrast analyses (Vul & Pashler, 2012). These analyses did not examine age effects, as this was the focus of the individual differences analyses using theoretically defined VOIs.

BART

Reward balloons versus control balloons. Average activation differences for reward versus control balloon trials resulted in regional activation and deactivation patterns that were comparable to results reported in previous neuroimaging analyses (Rao et al., 2008; Schonberg et al., 2012; Yu et al., 2016) (Figure B5, top panel). These included increased activation in ventral striatum, anterior insular cortex, and anterior cingulate cortex, as well as decreased activation in (ventromedial) prefrontal cortex (corrected using peak-level family-wise error correction, $p<0.05$).

Parametric risk. When we compared parametric modulation of activation differences as a function of the number of pumps (i.e., number of balloon in current trial) on reward versus control balloons, we also obtained results in line with previous findings (Schonberg et al., 2012; Tisdall et al., 2020; Yu et al., 2016) (Figure B5, second panel from the top). After controlling for multiple comparisons (via peak-level family-wise error correction, $p<0.05$), we specifically found that anterior insula activation differences parametrically track number of pumps; the more pumps are administered on a given trial, the higher the insula activation differences. Similar patterns were observed for nucleus accumbens and (ventromedial) prefrontal cortex, as well as anterior cingulate. As such, both the average reward versus control balloon contrast and the parametric modulation contrast pointed towards the involvement of a (largely overlapping) set of cortical and subcortical brain regions when participants made decisions under uncertainty in the BART. The regions we observed to show

activation differences in both BART contrasts are also in line with candidate regions postulated to be sources for age effects by the AIM framework (Samanez-Larkin & Knutson, 2015), and thus are also regions we targeted with our VOI analyses.

Linear versus exponential rewards. The originality of the reward balloon conditions implemented in this study precluded us from making comparative statements regarding activation patterns observed in the current and in previous work. In contrast to the other two BART analyses, none of the observed voxel-wise activation differences survived correction procedures (i.e., peak-level FWE correction, $p>0.05$); on average, when controlling for multiple comparisons, we found no activation differences between reward balloons following a linear versus exponential reward function. At the level of uncorrected ($p<0.001$) voxel-wise activation differences (Figure B5, third panel from the top), we found increased (ventromedial) prefrontal cortex activation for linear versus exponential balloons, as well as a few clusters of increased activation in medial parietal cortical regions, including bilateral precuneus, as well as more lateral parietal regions (e.g., angular gyrus). However, given that none of these regional activation differences survived correction procedures, it would be premature to conclude involvement of these regions for the contrast of interest. Moreover, the motivation for contrasting the different reward functions in the current analytical context was to examine whether the introduction of the exponential reward balloon would lead to different behavioral patterns (in particular for pumping and explosions), but behavioral analyses (Figure 3, Table B1) yielded no evidence for behavioral differences on the two reward balloons. We discuss potential reasons for the indifference between the two balloon types, which could equally account for the lack of neural differences between linear and exponential reward balloons.

Delay Discounting

Immediacy. Following previous work, we first contrasted trials that included an immediate option with trials that did not include an immediate option (Eppinger et al., 2012; McClure et al., 2004; Samanez-Larkin et al., 2011). When examining voxel-wise activation differences that were not corrected for multiple comparisons (Figure B5,

second panel from the bottom), we obtained main effects of increased activation in brain regions similar to those reported previously (Eppinger et al., 2012; McClure et al., 2004; Samanez-Larkin et al., 2011), including middle frontal gyrus, medial prefrontal cortex and medial orbitofrontal cortex, anterior cingulate gyrus, inferior frontal gyrus, bilateral anterior insula, bilateral caudate, as well as in more regionally extensive clusters including posterior cingulate cortex, precuneus and angular gyrus. However, mainly posterior regions including posterior cingulate gyrus, precuneus, and angular gyrus survived corrected for multiple comparisons (peak-level FWE, $p<0.05$) at the level of the whole brain. We also obtained a bilateral cluster of activation decreases for immediate versus delayed option trials in the occipital pole (peak-level FWE, $p<0.05$; not shown). To summarize, we did not find the same level of strong group-level activation differences in frontal and (ventral) striatal regions implicated in the processing of immediate choices found in the literature (Eppinger et al., 2012; McClure et al., 2004; Samanez-Larkin et al., 2011), despite choice-compatible incentivization.

Delay. We ran a final contrast to examine whether the brain tracks the actual length of the delay, i.e., whether a difference between the sooner and later option of four weeks may be processed differently (e.g., as less rewarding) compared to trials involving a shorter delay of two weeks. While we distinguished between trials with and without an immediate option for the first contrast analysis, for the delay contrast we did not distinguish trials based on the respective starting points, and included 32 trials which included options that were two weeks apart in one onset vector (regressor), and specified another onset vector for the 32 trials that included a four-week delay between the sooner and later option. Assessing patterns of uncorrected activation differences (Figure B5, bottom panel), we observed a few small clusters of voxels with increased activation for two- relative to four-week delays, including in bilateral putamen, bilateral superior temporal gyrus, and left central operculum. We also found clusters with decreased activation patterns, most prominently in bilateral superior frontal gyrus. However, none of the increased and decreased activation differences observed for this contrast survived correction methods (peak-level FWE, $p>0.05$).

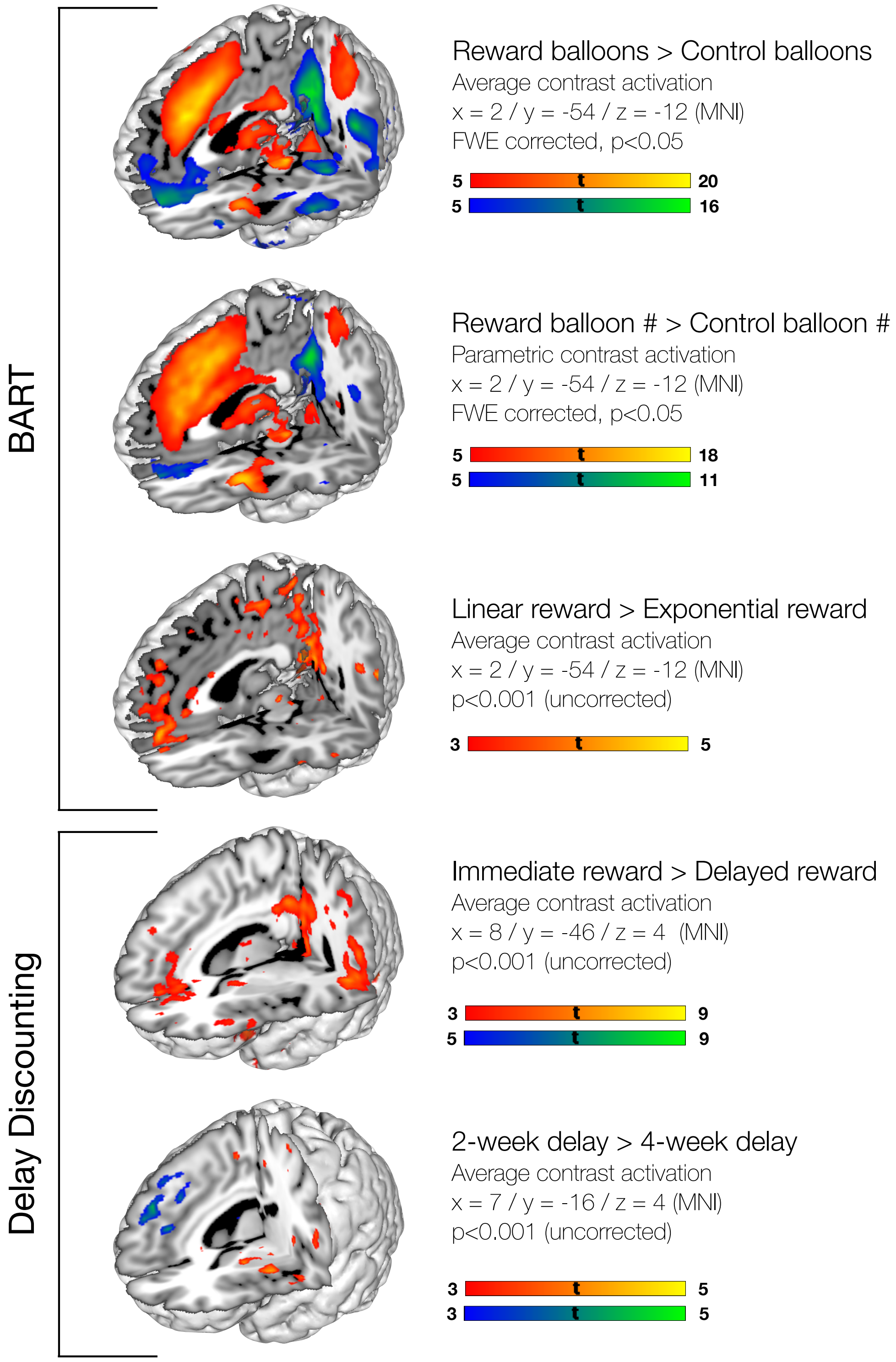

Figure B5

Group-level whole brain contrast activation maps for neural correlates of decision-making under uncertainty (N=175).

Bivariate associations

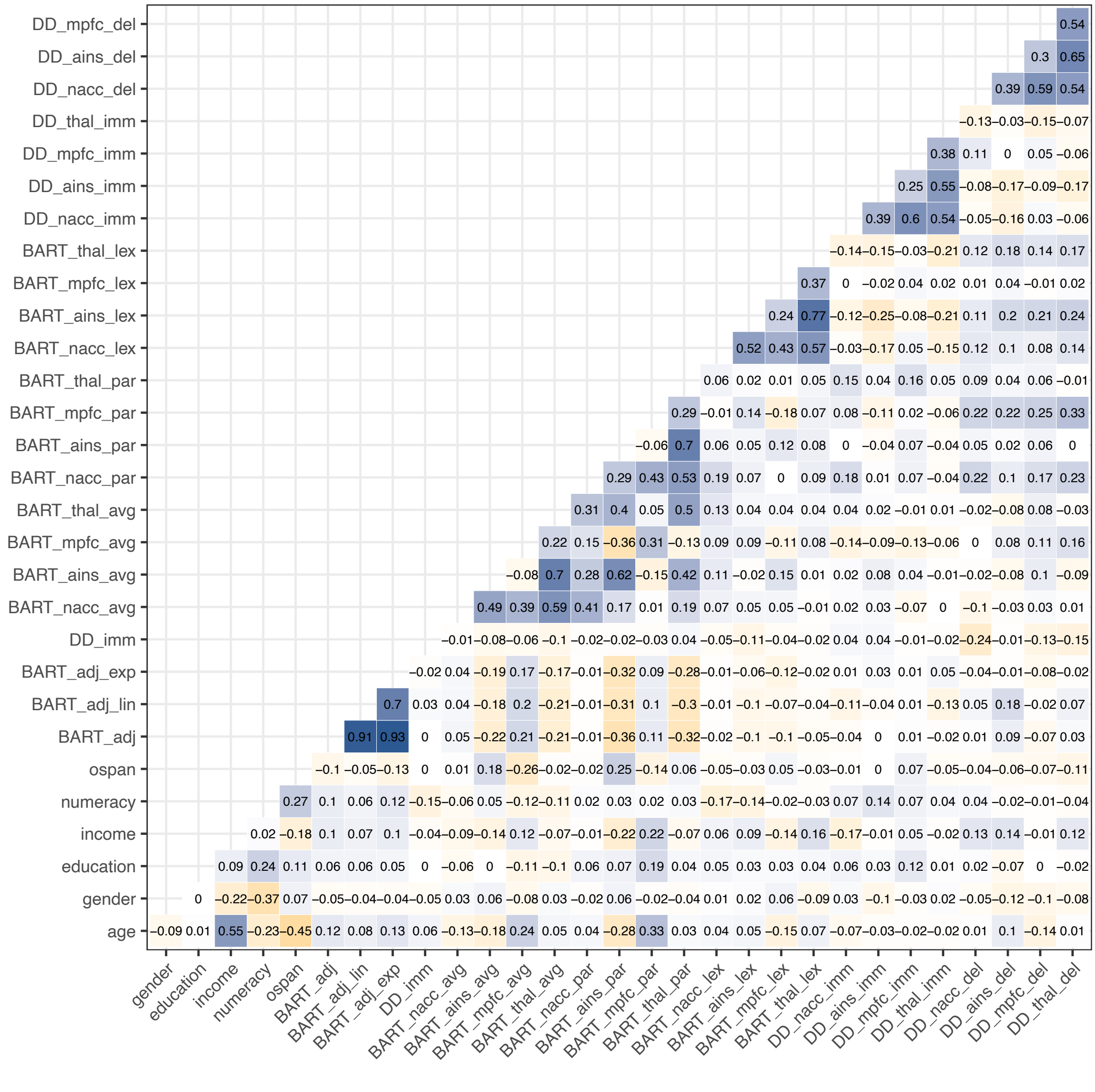

Figure B6

Correlation matrix of Pearson correlation coefficients for associations between all study variables.

Permutation testing

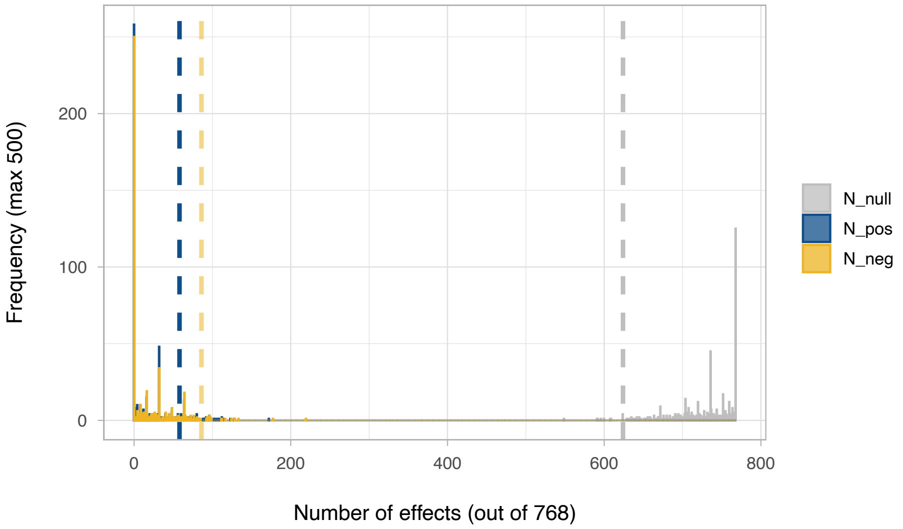

Figure B7

Summary of permutation testing analysis. The majority of SCAs on the shuffled data sets did not yield more significant (positive or negative) effects than observed in the unshuffled data, but yielded more null effects than observed in the unshuffled data. Note: N_null, N_pos, N_neg = null, positive, and negative age effects obtained from the 500 shuffled data sets; dotted lines represent the number of null, positive, and negative age effects observed in the unshuffled data.

References

Desikan, R. S., Ségonne, F., Fischl, B., Quinn, B. T., Dickerson, B. C., Blacker, D., Buckner, R. L., Dale, A. M., Maguire, R. P., Hyman, B. T., Albert, M. S., & Killiany, R. J. (2006). An automated labeling system for subdividing the human cerebral cortex on MRI scans into gyral based regions of interest. *NeuroImage*, *31*(3), 968–980. https://doi.org/10.1016/j.neuroimage.2006.01.021

Eppinger, B., Nystrom, L. E., & Cohen, J. D. (2012). Reduced Sensitivity to Immediate Reward during Decision-Making in Older than Younger Adults. *PLoS ONE*, *7*(5), 10.

Frey, R., Richter, D., Schupp, J., Hertwig, R., & Mata, R. (2021). Identifying robust correlates of risk preference: A systematic approach using specification curve analysis. *Journal of Personality and Social Psychology*, *120*(2), 538–557. https://doi.org/10.1037/pspp0000287

Lejarraga, T., Frey, R., Schnitzlein, D. D., & Hertwig, R. (2019). No effect of birth order on adult risk taking. *Proceedings of the National Academy of Sciences*, *116*(13), 6019–6024. https://doi.org/10.1073/pnas.1814153116

McClure, S. M., Laibson, D. I., Loewenstein, G., & Cohen, J. D. (2004). Separate neural systems value immediate and delayed monetary rewards. *Science*, *306*(5695), 503–507. https://doi.org/10.1126/science.1100907

Orben, A., & Przybylski, A. K. (2019). The association between adolescent well-being and digital technology use [ISBN: 4156201805 Publisher: Springer US]. *Nature Human Behaviour*, *3*(2), 173–182. https://doi.org/10.1038/s41562-018-0506-1

Rao, H., Korczykowski, M., Pluta, J., Hoang, A., & Detre, J. A. (2008). Neural correlates of voluntary and involuntary risk taking in the human brain: An fMRI Study of the Balloon Analog Risk Task (BART) [ISBN: 1053-8119]. *NeuroImage*, *42*(2), 902–910. https://doi.org/10.1016/j.neuroimage.2008.05.046

Rohrer, J. M., Egloff, B., & Schmukle, S. C. (2017). Probing Birth-Order Effects on Narrow Traits Using Specification-Curve Analysis. *Psychological Science*, *28*(12), 1821–1832. https://doi.org/10.1177/0956797617723726

Sacchet, M. D., & Knutson, B. (2013). Spatial smoothing systematically biases the localization of reward-related brain activity [Publisher: Elsevier Inc.]. *NeuroImage*, *66*, 270–277. https://doi.org/10.1016/j.neuroimage.2012.10.056

Samanez-Larkin, G. R., & Knutson, B. (2015). Decision making in the ageing brain: Changes in affective and motivational circuits. *Nature Reviews Neuroscience*, *16*(May). https://doi.org/10.1038/nrn3917

Samanez-Larkin, G. R., Mata, R., Radu, P. T., Ballard, I. C., Carstensen, L. L., & McClure, S. M. (2011). Age differences in striatal delay sensitivity during intertemporal choice in healthy adults [ISBN: 1662-453X (Electronic)\r1662-453X (Linking)]. *Frontiers in Neuroscience*, *5*(NOV), 1–12. https://doi.org/10.3389/fnins.2011.00126

Schonberg, T., Fox, C. R., Mumford, J. A., Congdon, E., Trepel, C., & Poldrack, R. A. (2012). Decreasing ventromedial prefrontal cortex activity during sequential risk-taking: An FMRI investigation of the balloon analog risk task [ISBN: 1662-453X (Electronic)\r1662-453X (Linking)]. *Frontiers in Neuroscience*, *6*(June), 1–11. https://doi.org/10.3389/fnins.2012.00080

Seaman, K. L., Brooks, N., Karrer, T. M., Castrellon, J. J., Perkins, S. F., Dang, L. C., Hsu, M., Zald, D. H., & Samanez-Larkin, G. R. (2018). Subjective value representations during effort, probability and time discounting across adulthood. *Social Cognitive and Affective Neuroscience*, *13*(5), 449–459. https://doi.org/10.1093/scan/nsy021

Steegen, S., Tuerlinckx, F., Gelman, A., & Vanpaemel, W. (2016). Increasing Transparency Through a Multiverse Analysis. *Perspectives on Psychological Science*, *11*(5), 702–712. https://doi.org/10.1177/1745691616658637

Tisdall, L., Frey, R., Horn, A., Ostwald, D., Horvath, L., Pedroni, A., Rieskamp, J., Blankenburg, F., Hertwig, R., & Mata, R. (2020). Brain-outcome associations for risk taking depend on the measures used to capture individual differences. *Frontiers in Behavioral Neuroscience*, *14*(November). https://doi.org/10.31234/osf.io/3sc9j

Vul, E., & Pashler, H. (2012). Voodoo and circularity errors [ISBN: 1053-8119 Publisher: Elsevier Inc.]. *NeuroImage*, *62*(2), 945–948. https://doi.org/10.1016/j.neuroimage.2012.01.027

Yu, J., Mamerow, L., Lei, X., Fang, L., & Mata, R. (2016). Altered Value Coding in the Ventromedial Prefrontal Cortex in Healthy Older Adults. *Frontiers in Aging Neuroscience*, *8*. https://doi.org/10.3389/fnagi.2016.00210
